# Phenotype-based clusters, inflammation and cardiometabolic complications in older people before the diagnosis of type 2 diabetes: KORA F4/FF4 cohort study

**DOI:** 10.1186/s12933-025-02617-8

**Published:** 2025-02-19

**Authors:** Marie-Theres Huemer, Maria C. Spagnuolo, Haifa Maalmi, Robert Wagner, Gidon J. Bönhof, Margit Heier, Wolfgang Koenig, Wolfgang Rathmann, Katsiaryna Prystupa, Jana Nano, Dan Ziegler, Annette Peters, Michael Roden, Barbara Thorand, Christian Herder

**Affiliations:** 1 Institute of Epidemiology, Helmholtz Zentrum München, German Research Center for Environmental Health (GmbH), Neuherberg, Germany; 2 Institute for Clinical Diabetology, German Diabetes Center (DDZ), Leibniz Center for Diabetes Research at Heinrich Heine Universität, Auf’m Hennekamp 65, 40225 Düsseldorf, Germany; 3 German Center for Diabetes Research (DZD), Partner Düsseldorf, Neuherberg, Germany; 4 Department of Endocrinology and Diabetology, Medical Faculty and University Hospital Düsseldorf, Heinrich Heine Universität, Düsseldorf, Germany; 5 School of Medicine and Health, German Heart Centre, TUM University Hospital, Technical University of Munich, Munich, Germany; 6 German Centre for Cardiovascular Research (DZHK), Partner Site Munich Heart Alliance, Munich, Germany; 7 Institute of Epidemiology and Medical Biometry, University of Ulm, Ulm, Germany; 8 Institute for Biometrics and Epidemiology, German Diabetes Center (DDZ), Leibniz Center for Diabetes Research at Heinrich Heine Universität, Düsseldorf, Germany; 9 German Center for Diabetes Research (DZD), Partner München-Neuherberg, Neuherberg, Germany; 10 Institute for Medical Information Processing, Biometry and Epidemiology (IBE), Faculty of Medicine, Pettenkofer School of Public Health, LMU Munich, Munich, Germany

**Keywords:** Cardiovascular disease, Chronic kidney disease, Cohort, Diabetes, Heterogeneity, Inflammation, Mortality, Polyneuropathy, Prediabetes, Subtypes

## Abstract

**Background:**

Using a data-driven approach, six clusters with different risk profiles and burden of complications were recently identified in middle-aged people before the diagnosis of type 2 diabetes (T2D). We aimed to investigate whether these clusters could be generalised to older people and if subclinical inflammation was related to their cardiometabolic risk profiles.

**Methods:**

We assigned 843 participants of the KORA F4 study aged 61–82 years without T2D to the six previously defined phenotype-based clusters. Based on 73 biomarkers of subclinical inflammation, we derived an inflammation-related score (“inflammatory load”) using principal component analysis to assess subclinical inflammation. Risk factors, inflammatory load as well as prevalence and incidence of (pre)diabetes-related complications were compared between the clusters using pairwise comparisons and regression analyses.

**Results:**

Clusters 1 and 2 had the lowest cardiometabolic risk, whereas clusters 5 and 6 the highest. T2D risk was highest in clusters 3, 4, 5, and 6 compared with the low-risk cluster 2 (age- and sex-adjusted ORs between 3.6 and 34.0). In cross-sectional analyses, there were significant between-cluster differences in chronic kidney disease (CKD), distal sensorimotor polyneuropathy (DSPN) and cardiovascular disease (all p < 0.045). In prospective analyses (mean follow-up time 6.5–8.3 years), clusters differed significantly in CKD and DSPN incidence, but not in incident CVD or all-cause mortality. The inflammatory load was highest in the high-risk cluster 5 and lowest in cluster 2. Adjustment for the inflammatory load had only a minor impact on the aforementioned differences in outcomes between clusters.

**Conclusions:**

Our findings extend the knowledge about the previously identified six phenotype-based clusters in older people without T2D. Differences between clusters were more pronounced for T2D risk than for prevalent or incident (pre)diabetes-related complications and absent for mortality. The high cardiometabolic risk corresponded to the high inflammatory load in cluster 5 but not to the lower inflammatory load of high-risk clusters 3 and 6.

**Graphical abstract:**

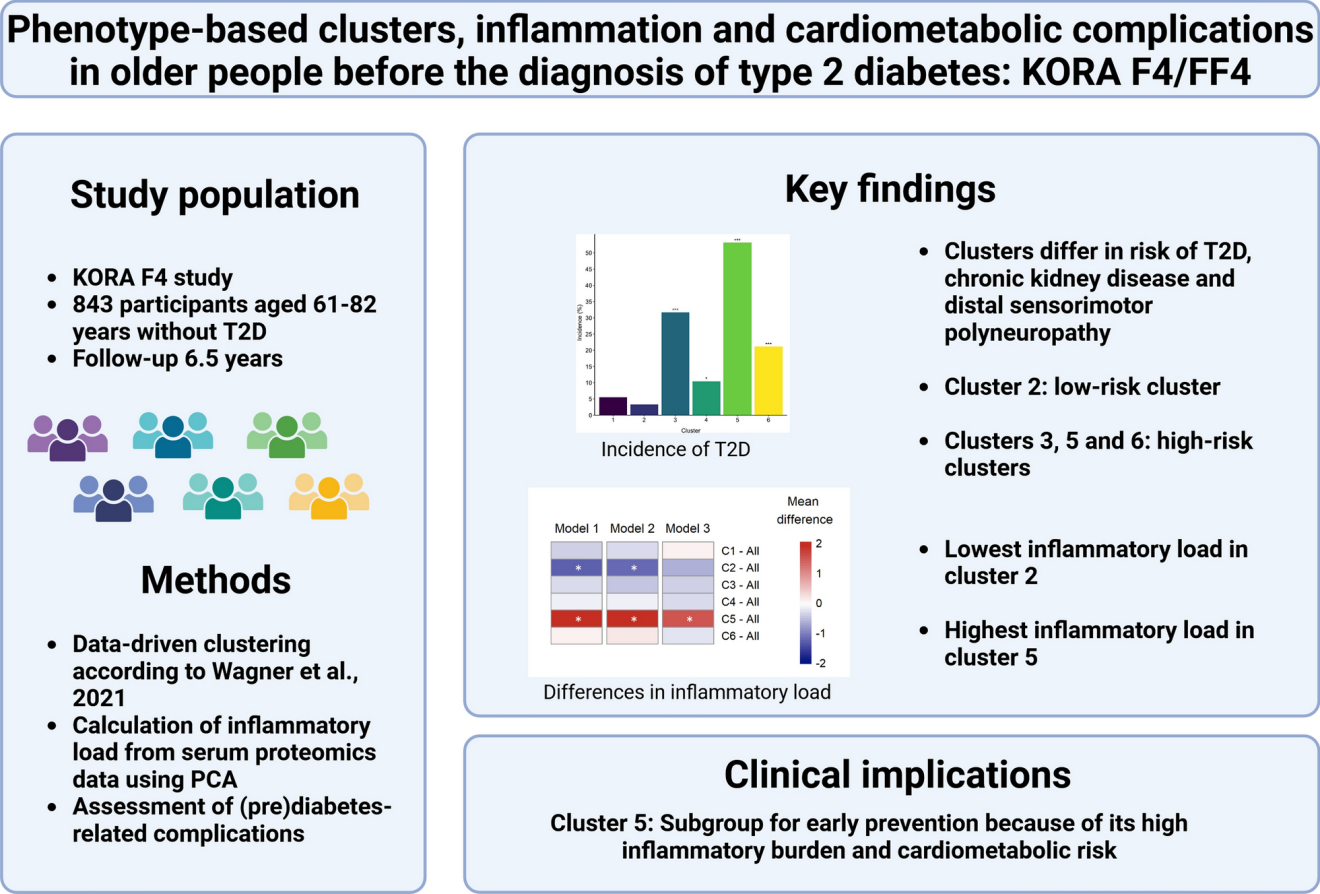

**Supplementary Information:**

The online version contains supplementary material available at 10.1186/s12933-025-02617-8.

## Background

The heterogeneity of diabetes has been addressed by proposing novel subtypes of diabetes [1, 2]. The most widely replicated approach identified—in addition to severe autoimmune diabetes (SAID) mainly reflecting type 1 diabetes (T1D)—four subtypes reflecting type 2 diabetes (T2D) that were designated severe insulin-deficient diabetes (SIDD), severe insulin-resistant diabetes (SIRD), mild obesity-related diabetes (MOD) and mild age-related diabetes (MARD) [3, 4]. These subtypes display distinct patterns of clinical features and disease progression [1–3]. Their differences in the incidence of diabetes-related complications may partly be attributable to differences in age, anthropometric and metabolic characteristics, while these subtypes also differ in biomarkers of inflammation and immune cells. Among the five novel subtypes, SIRD is characterised both by the most pronounced proinflammatory profile [5, 6] and the highest risk for diabetes-related complications [3]. Given the development of T2D over decades it is likely that such heterogeneity is already present before the diagnosis of diabetes, e.g. in people with prediabetes or in older people with a high T2D risk.

Prediabetes and older age are not only high-risk states for the incidence of type 2 diabetes but also associated with a higher risk for “(pre)diabetes-related” complications and mortality compared to normal glucose tolerance [7, 8]. A recent study described six novel subphenotypes/clusters in people before the diagnosis of diabetes that differ in clinical and metabolic characteristics and in the risk of (pre)diabetes-related complications [9]. The initial cluster analysis relied on highly sophisticated phenotyping but a replication of the clusters was possible using variables that are more commonly available [9]. Importantly, these six clusters were derived from middle-aged populations, and it is unknown if they are generalisable to older populations with higher cardiometabolic risk. Additionally, the clusters differed in circulating high-sensitivity C-reactive protein (hsCRP) [9] with the highest hsCRP levels in cluster 5 (“high risk with insulin-resistant fatty liver”) and cluster 6 (“high risk with visceral fat and nephropathy”). Of note, both clusters were among the ones with the highest risk for complications and mortality [9]. However, a more comprehensive assessment of subclinical inflammation in the different clusters based on multiple biomarkers that may explain differences in disease progression has not been performed.

Therefore, our study aimed (i) to replicate the clusters described by Wagner et al. [9] in older adults from the population-based cohort study Cooperative Health Research in the Region of Augsburg (KORA) F4/FF4, (ii) to characterise differences between the clusters in incident T2D, (iii) to evaluate differences in the prevalence and incidence of (pre)diabetes-related complications (chronic kidney disease [CKD], distal sensorimotor polyneuropathy [DSPN], cardiovascular disease [CVD]) and mortality and (iv) to assess differences in > 70 biomarkers of subclinical inflammation and their pooled inflammatory load between the clusters.

## Methods

### Study design and population

This study was based on data from the KORA F4 study (2006–2008) and the KORA FF4 study (2013–2014), both follow-up examinations of the population-based KORA S4 study (1999–2001) conducted in Augsburg and two adjacent counties in Germany [10].

From the 3,080 participants of the KORA F4 study, we excluded participants who withdrew consent (n = 5), were not in the age range of 61–82 years (n = 1915), had clinically diagnosed or OGTT-diagnosed diabetes at F4 (n = 254) or had missing information on the diabetes status (n = 25) and/or had missing information on the prediabetes phenotyping variables (n = 14). After these exclusions, 867 participants remained for assignment to prediabetes clusters. Of note, the restriction to the age range between 61 and 82 years was necessary because data for 2-h insulin (OGTT), which is essential for the clustering, are only available in this subgroup. We additionally excluded participants with missing information on biomarkers of inflammation (n = 24) leaving 843 participants for complete-case analysis investigating the inflammatory load. All 843 participants had fasted for at least 8 h before blood sampling. Supplementary Fig. 1, Additional file 1 describes these exclusions in detail. For the prospective analyses on incident T2D, CKD and DSPN (mean follow-up time 6.5 ± 0.2 years), we further excluded participants who did not participate in FF4. CVD incidence and all-cause mortality were assessed until the end of 2016 (mean follow-up time 7.8 ± 1.9 years and 8.3 ± 1.7 years for incident CVD and all-cause mortality, respectively). Participants lost-to-follow-up were censored at the time of last information.

### Assessment of variables for the cluster assignment

Cluster assignment was performed as described for the Whitehall II cohort [9; see also https://cluster.apps.dzd-ev.org/] based on the following variables in KORA F4: age, BMI, waist circumference, hip circumference, fasting glucose, 2-h glucose (OGTT), fasting insulin, 2-h insulin (OGTT), fasting triglycerides, fasting HDL-cholesterol, insulin secretion (Stumvoll) and insulin sensitivity (Matsuda). This led to the following six clusters: cluster 1, Low risk; cluster 2, Very low risk; cluster 3, Beta-cell failure; cluster 4, Low risk obese; cluster 5, High risk insulin-resistant fatty liver; cluster 6, High risk visceral fat nephropathy.

Anthropometric and metabolic variables were measured as described [11, 12]. Data from the OGTT were used to assess glucose tolerance categories (normal glucose tolerance [NGT], impaired fasting glucose [IFG], impaired glucose tolerance [IGT]) based on the 1999 World Health Organization diagnostic criteria [11]. Insulin secretion was assessed using Stumvoll’s first-phase insulin secretion index based on fasting and 2-h glucose and insulin levels [13]. Insulin sensitivity was quantified using the whole-body insulin sensitivity index (ISI(composite)) as described by Matsuda and DeFronzo [14].

### Assessment of T2D, (pre)diabetes-related complications and mortality

Prevalent T2D at KORA F4 was defined based on a validated physician diagnosis or as newly diagnosed diabetes by OGTT (≥ 7.0 mmol/l fasting or ≥ 11.1 mmol/l 2-h glucose) in KORA F4. Incident T2D was defined based on a validated physician diagnosis between KORA F4 and FF4 or as newly diagnosed diabetes by OGTT (≥ 7.0 mmol/l fasting or ≥ 11.1 mmol/l 2-h glucose) in KORA FF4 [11].

Kidney function was assessed using the estimated glomerular filtration rate (eGFR, ml/min/1.73m^2^) which was calculated according to the Chronic Kidney Disease Epidemiology (CKD-EPI 2012) equation based on both serum creatinine and cystatin-C. An eGFR < 60 ml/min/1.73m^2^ was used to define prevalent and incident CKD at KORA F4 or FF4.

DSPN was assessed using the physical examination component of the Michigan Neuropathy Screening Instrument (MNSI) as described [15]. Prevalent and incident DSPN were defined using a cut-off at > 3 points based on the examinations at F4 and FF4.

Prevalent CVD included myocardial infarction (MI) and stroke at F4 based on self-reported information and data from the Augsburg MI registry if applicable. Incident CVD includes a combined endpoint of incident non-fatal and fatal MI including all coronary heart deaths as well as incident non-fatal and fatal stroke (ischaemic and haemorrhagic strokes without transient ischaemic attacks).

Vital status was determined using population registries, and death certificates were requested from the local health authorities to ascertain causes of death. In addition to the follow-up examination FF4 we used data from follow-up questionnaires sent to all reachable participants in 2008–2009 and in 2016 to obtain information on the occurrence of incident non-fatal MI and stroke. All incident events were validated by hospital discharge records, information from the treating physician or data from the Augsburg MI registry if coronary events occurred in the age range (≤ 74 years until 2009 and ≤ 84 years thereafter) and the area covered by the MI registry.

### Biomarkers of subclinical inflammation

Biomarkers of subclinical inflammation were measured in fasting serum using proximity extension assay technology (Target 96 Inflammation panel, OLINK Proteomics, Uppsala, Sweden) in KORA F4 participants aged 61–82 years as described [16]. This proximity extension assay allows the measurement of 92 protein biomarkers including pro- and anti-inflammatory cytokines, chemokines, growth factors and factors involved in acute inflammatory and immune responses, angiogenesis, fibrosis and endothelial activation. These biomarkers are designated “biomarkers of inflammation” although some of them may also be considered metabolic biomarkers or biomarkers also reflecting other pathways. Biomarker levels are given as normalised protein expression (NPX) values, which are comparable in their distribution to log2-transformed protein concentrations. Out of the 92 measured proteins, 21 biomarkers were excluded due to quality control issues (≥ 25% of values below the limit of detection and/or inter-assay coefficient of variation > 20%) [16]. Remaining values below the limit of detection were retained in the data and not substituted. We examined each individual biomarker for outliers and set one implausibly low value of one participant for transforming growth factor beta-1 proprotein (TGFb1) to the next lowest available value of the protein.

In addition, we included hsCRP and tumour necrosis factor alpha (TNFα), which were measured separately. hsCRP was measured in EDTA plasma using high-sensitivity latex-enhanced nephelometric assay on a BN II analyzer (Dade Behring) and TNFα was measured in serum by ELISA [17]. hsCRP and TNFα levels were log_2_ transformed as their values were not normally distributed. Therefore, the final data set consisted of 73 biomarkers of subclinical inflammation, which were all transformed using z-standardisation.

### Additional participant characteristics

Systolic and diastolic blood pressure were measured according to standardised protocols. Hypertension was defined as blood pressure of 140/90 mmHg or higher or by the use of antihypertensive medication given that participants were aware of being hypertensive [18]. Information on medication was collected by trained medical interviewers. Being physically active was defined as exercising in summer and winter for more than 1 h/week [19].

### Statistical analysis

Cluster assignment was based on the aforementioned variables and performed using the same method as in the Whitehall II cohort [9]. Summary statistics are reported as means ± SD for continuous variables and percentages for categorical variables. Differences between the clusters were calculated using one-way ANOVA for continuous variables and chi-square test or Fisher’s exact test (used for cell sizes < 5) for categorical variables. Chi-square test or Fisher’s exact test were also used to assess differences in prevalent and/or incident outcomes (T2D, CKD, DSPN) among the clusters as appropriate, and for pairwise comparisons between clusters. Comparisons between clusters were performed with cluster 2 as the reference since this cluster had the lowest inflammatory load (see below for the calculation) compared to the other clusters.

We performed principal component analysis (PCA) with all 73 biomarkers of inflammation using the R package “FactoMineR” [20] to derive a score of “inflammatory load” for each participant. The “inflammatory load” was derived by combining levels of multiple biomarkers and indicates a state of subclinical inflammation (higher scores indicate a higher inflammation). After conducting the PCA, we assessed the stability of the first five principal components by bootstrapping of the PCA. Hereby, the Pearson correlation coefficient was calculated between the variable scores before bootstrapping (original sample) and each of the 1000 bootstrap samples individually. Boxplots were used to visualise the median correlation coefficient of all 1000 coefficients and their distribution. We then calculated the inflammatory load, i.e. the principal component score, of each participant from principal component 1. This approach was based on the analysis strategy of Morrisette-Thomas et al. [21]. Afterwards, we compared the inflammatory load and the individual levels of the multiple biomarkers of subclinical inflammation between the clusters using boxplots and pairwise comparisons between all clusters (15 comparisons) and between each cluster against all other clusters combined (6 comparisons). For the comparisons of the inflammatory load between the clusters, the following three models were used: Model 1, unadjusted; model 2, adjusted for age and sex; model 3, adjusted for age, sex, and BMI. Pairwise comparisons were performed using the R package “multcomp” [22]. In addition, Spearman’s rank correlation was applied to calculate the correlation between the inflammatory load and the clustering variables.

The associations of the six clusters with prevalent CKD, DSPN and CVD as well as with incident T2D, CKD and DSPN were assessed with logistic regression to obtain odds ratios (OR) and 95% confidence intervals (CI). The associations of clusters with incident CVD and all-cause mortality were assessed with Cox regression to obtain hazard ratios (HR) and 95% CI as the times of events were known during the follow-up period. Additionally, for incident CVD and all-cause mortality, which are time-to-event outcomes, we plotted the Kaplan–Meier survival curves stratified by cluster and used the log-rank test to assess differences between survival time. For both the logistic regression and Cox regression analyses four models were built. Model 1 was unadjusted, model 2 was adjusted for age and sex, model 3 was adjusted for age, sex and BMI and model 4 was adjusted for age, sex and inflammatory load.

Statistical analyses were performed using R version 4.3.1 and SAS statistical software (version 9.4; SAS Institute Inc., Cary, NC, USA). A *p* value of < 0.05 was used to indicate statistical significance. Benjamini–Hochberg adjusted *p* values were additionally calculated to account for multiple testing.

## Results

### Baseline sample characteristics

We replicated six clusters among older adults without T2D in the KORA study. Among the clusters, there were significant differences between all clustering variables except for age (all other *p* < 0.05, Table 1). Obesity indices were higher in clusters 4, 5 and 6 compared to clusters 1, 2 and 3. Glucose levels were highest in clusters 3, 5 and 6, which were also characterised by the lowest insulin sensitivity. Insulin secretion was lowest in clusters 2, 3 and 4. Lipid levels were most unfavourable (high triglycerides, low HDL cholesterol) in cluster 5. Cluster 5 had the highest proportion of individuals with hypertension.

**Table 1:** Clinical characteristics and other risk factors of the clusters (n = 867)

	Cluster 1 (Low risk) (n = 87, 10.03%)	Cluster 2 (Very low risk) (n = 313, 36.10%)	Cluster 3 (Beta-cell failure) (n = 159, 18.34%)	Cluster 4 (Low risk obese) (n = 150, 17.30%)	Cluster 5 (High risk IR fatty liver) (n = 71, 8.19%)	Cluster 6 (High risk visceral fat nephropathy) (n = 87, 10.03%)	*p*
*Clustering variables*							
Age (years)	69.62 ± 5.53	69.58 ± 5.52	70.86 ± 5.36	69.82 ± 5.38	70.38 ± 5.00	69.37 ± 5.12	0.1678
BMI (kg/m^2^)	26.09 ± 1.84	25.04 ± 2.63	28.61 ± 2.73	30.60 ± 2.93	32.76 ± 3.55	32.35 ± 4.87	** < 0.0001**
Waist circumference (cm)	91.32 ± 6.76	88.77 ± 9.54	97.94 ± 8.92	102.03 ± 7.27	108.47 ± 8.99	106.76 ± 13.20	** < 0.0001**
Hip circumference (cm)	102.38 ± 4.06	101.61 ± 5.79	106.73 ± 5.82	112.06 ± 6.47	115.26 ± 8.83	114.20 ± 11.30	** < 0.0001**
Fasting glucose (mmol/l)	5.24 ± 0.41	5.19 ± 0.47	5.72 ± 0.53	5.32 ± 0.48	5.96 ± 0.56	5.52 ± 0.41	** < 0.0001**
2-h glucose (mmol/l)	6.29 ± 1.06	5.83 ± 1.49	8.57 ± 1.18	6.01 ± 1.04	8.71 ± 1.43	6.54 ± 1.11	** < 0.0001**
Fasting insulin (pmol/l)	50.59 ± 25.47	23.40 ± 13.03	43.66 ± 20.28	33.63 ± 13.56	283.50 ± 795.74	183.45 ± 310.23	** < 0.0001**
2-h insulin (pmol/l)	471.69 ± 214.30	209.11 ± 145.78	572.19 ± 240.51	309.61 ± 151.48	1242.98 ± 1356.0	788.42 ± 471.24	** < 0.0001**
Triglycerides (mg/dl)	134.35 ± 60.76	95.66 ± 48.87	138.63 ± 60.51	127.65 ± 54.94	199.38 ± 105.55	136.45 ± 50.22	** < 0.0001**
HDL cholesterol (mg/dl)	52.57 ± 9.38	65.97 ± 14.79	54.90 ± 11.24	53.14 ± 10.84	45.86 ± 9.13	52.35 ± 12.31	** < 0.0001**
Insulin secretion (Stumvoll)	947.77 ± 328.88	596.17 ± 268.88	570.98 ± 335.23	695.84 ± 225.27	2673.70 ± 6173.2	1995.40 ± 2307.3	** < 0.0001**
Insulin sensitivity (Matsuda)	18.26 ± 5.51	58.19 ± 68.15	15.41 ± 4.68	27.88 ± 9.74	7.22 ± 4.12	8.80 ± 3.91	** < 0.0001**
*Other clinical variables*							
Sex, Men (%)	47.13	52.40	50.31	40.67	38.03	55.17	0.0616
Systolic blood pressure (mmHg)	125.37 ± 20.71	126.90 ± 19.97	129.52 ± 19.15	126.34 ± 19.01	130.30 ± 19.48	126.04 ± 18.24	0.3680
Diastolic blood pressure (mmHg)	73.17 ± 10.17	73.80 ± 9.35	75.14 ± 10.57	74.09 ± 10.19	75.47 ± 10.45	74.28 ± 10.68	0.5564
Hypertension (%)	59.77	43.41	67.92	61.74	80.00	63.22	** < 0.0001**
Physically active (%)	55.17	64.95	44.65	46.62	49.30	43.68	** < 0.0001**
Lipid-lowering medication (%)	27.59	20.26	24.53	21.33	16.90	22.99	0.5650
NSAIDs (%)	1.15	5.13	2.52	4.00	2.82	3.45	0.5971

Cluster designations are based on Wagner et al. [9]

Data are given as mean ± SD. Significant differences between the six clusters (*p* < 0.05) are indicated by bold print

Missing values: NSAIDs (non-steroidal anti-inflammatory drugs; n = 1), lipid-lowering medication (n = 2), physical activity (n = 4) and hypertension (n = 4)

**Fig. 1:**
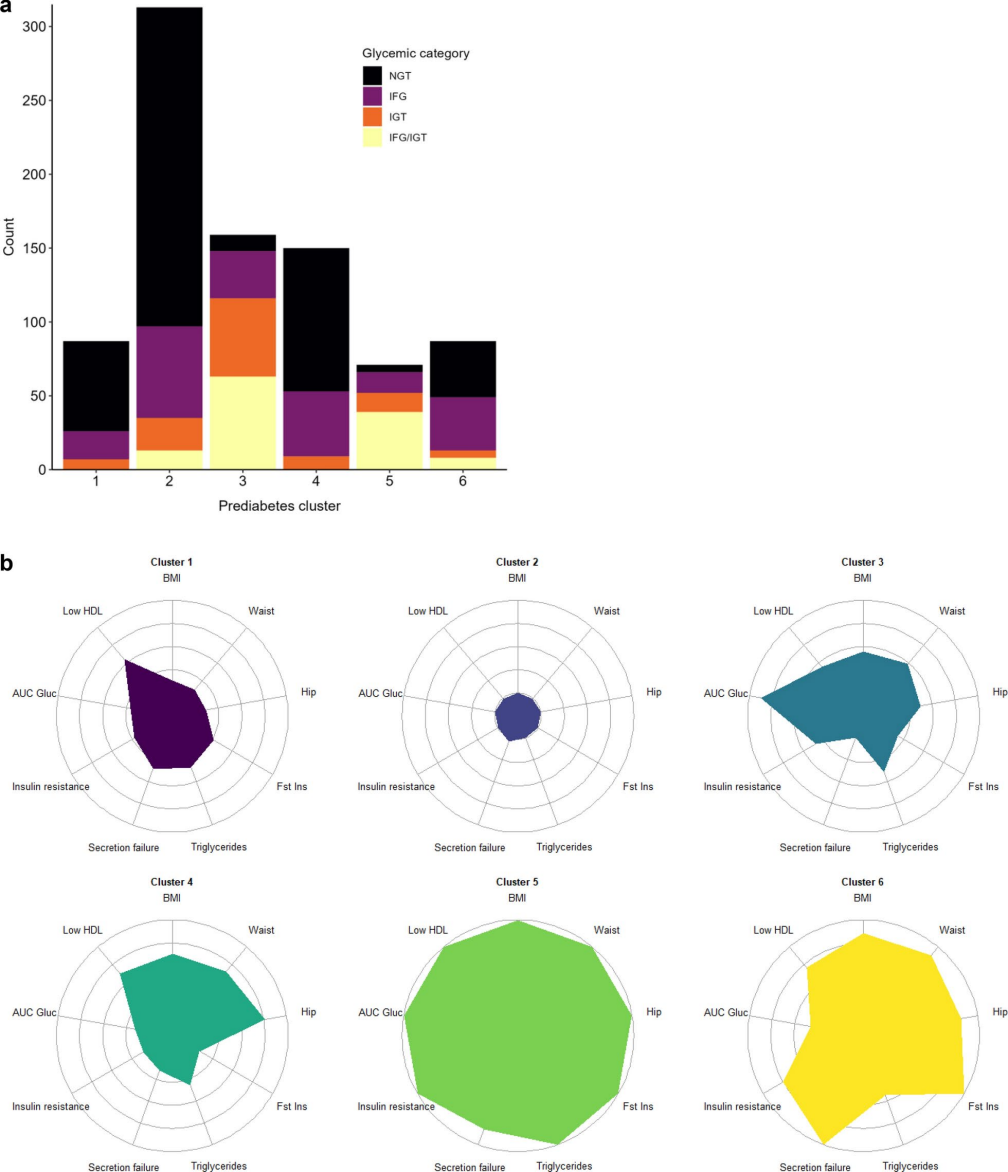
Glucose tolerance categories and of the clustering variables in the six clusters. **a** Distribution of participants by glucose tolerance category and cluster. **b** Distribution of the clustering variables within each cluster. Medians are plotted for each cluster with the corresponding standardised level (mean = 0, standard deviation = 1) for each variable. AUC Gluc, area under the glucose curve during OGTT (2-point glucose area-under-curve was calculated as 120 * ((fasting blood glucose in mmol/L + blood glucose at 2 h in mmol/L)/2); BMI, body mass index (kg/m2); Fst Ins, fasting insulin (µIU/ml); hip, Hip circumference (cm); Insulin resistance: 1 / insulin sensitivity (Matsuda’s index for estimating insulin sensitivity during OGTT was calculated as 10,000/sqrt (fasting blood glucose in mmol/L * fasting insulin in pmol/L* (fasting insulin in pmol/L + insulin at 2 h in pmol/L)/2 * (fasting blood glucose in mmol/L + blood glucose at 2 h in mmol/L)/2)); Low HDL, HDL cholesterol in mmol/L * -1 (directionally flipped so that a higher area in the figure indicates a higher cardiometabolic risk); Secretion failure, Stumvoll’s first phase insulin secretion index calculated as 2503 + 6.476 * fasting insulin in pmolL—126.5 * blood glucose at 2 h in mmol/L + 0.954 * insulin at 2 h in pmol/L—293.3 * fasting blood glucose in mmol/L; Waist, waist circumference (cm)

The distribution of individuals by glucose tolerance status is shown in Supplementary Table 1, Additional file 1 and Fig. 1a. Clusters 1, 2 and 4 had the largest proportion of individuals with NGT. Clusters 3, 5 and 6 had the largest proportion of individuals with IGT or IFG/IGT.

The radar charts in Fig. 1b visualise the distribution of cardiometabolic risk factors used for the cluster assignment. The size of the coloured areas indicates that clusters 1 and 2 had the most favourable and clusters 5 and 6 the most unfavourable distribution of risk factors.

### Incidence of T2D

**Fig. 2:**
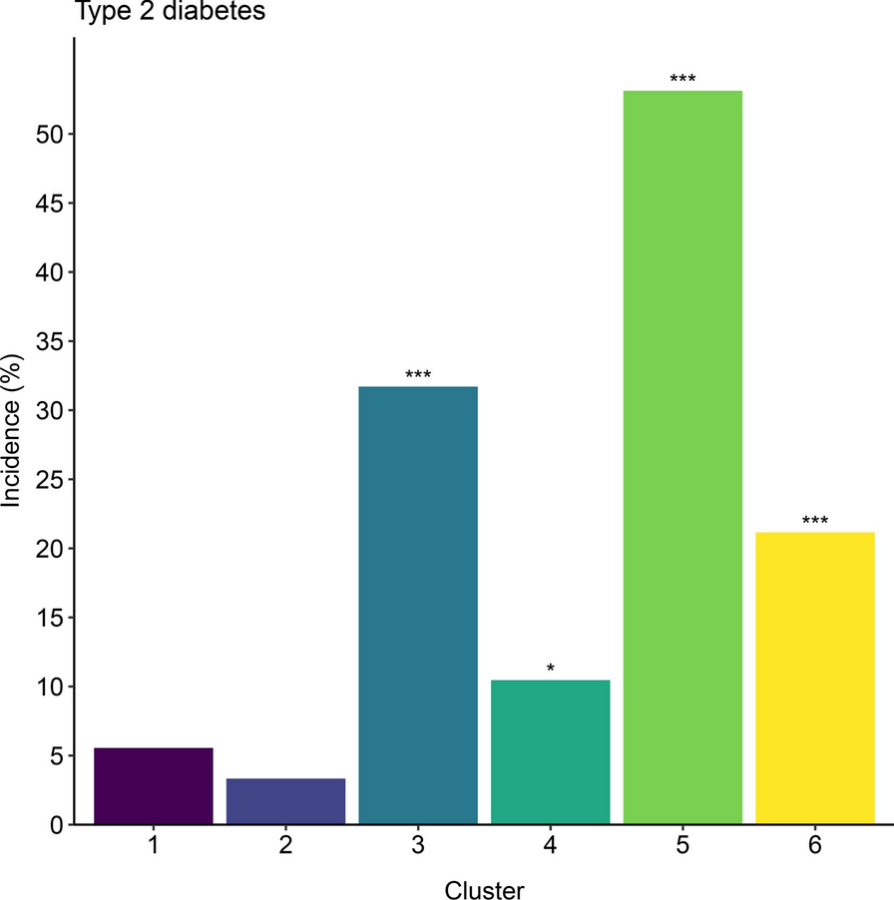
Incidence of T2D in the clusters. See also Supplementary Table 2, Additional file 1 for numbers of cases in each cluster. *p < 0.05, *** p < 0.001 for comparisons between each cluster to cluster 2 (reference)

The incidence of T2D differed significantly between the clusters (*p* < 0.0001; Fig. 2, Supplementary Table 2, Additional file 1). Using cluster 2 as reference (Supplementary Table 3, Additional file 1), T2D incidence was higher in clusters 3, 4, 5 and 6 after adjustment for age and sex (p_BH_ < 0.05). All but one of these associations remained significant after additional adjustment for BMI.

### Prevalence of (pre)diabetes-related complications among clusters

**Fig. 3:**
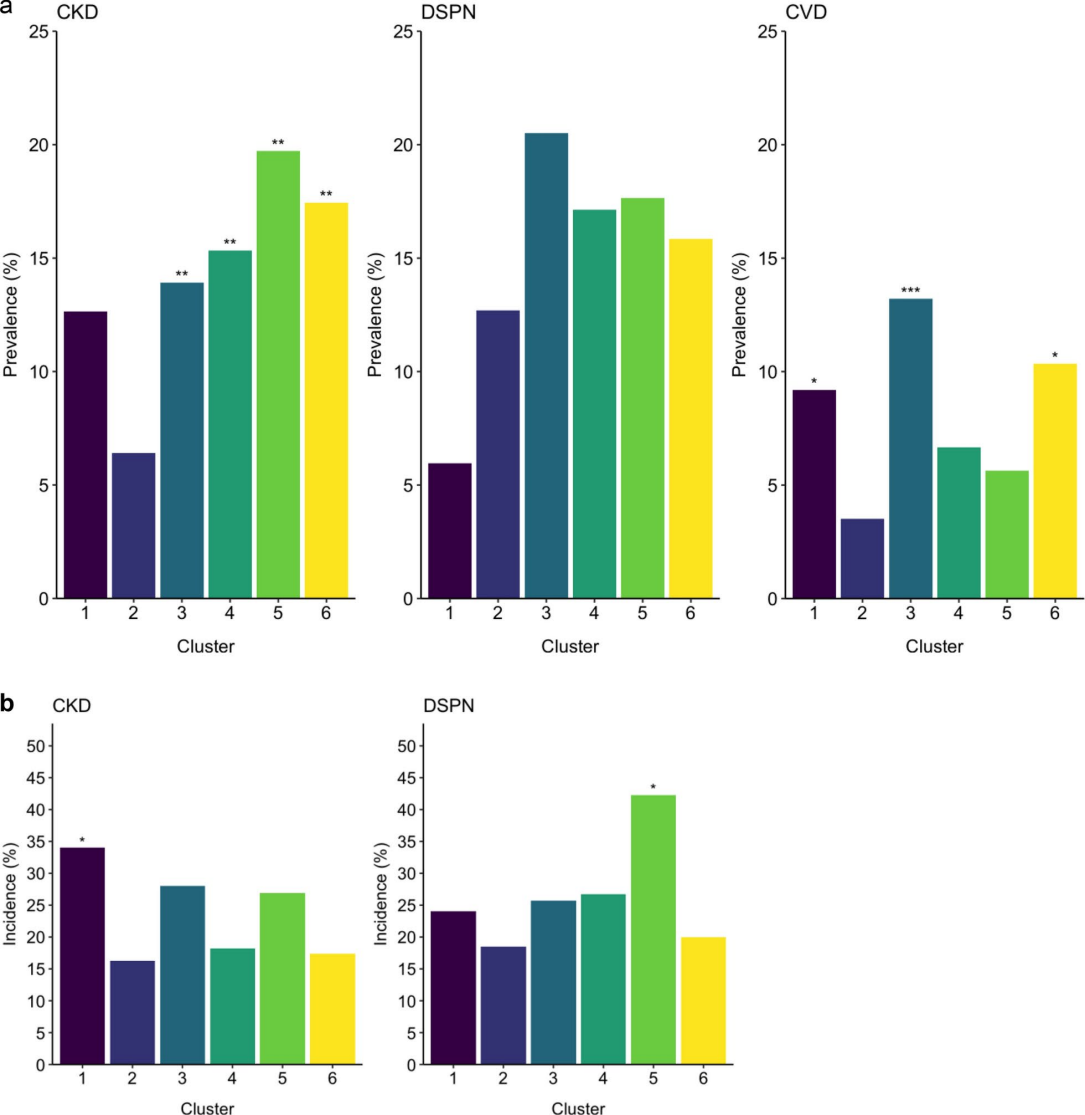
(Pre)diabetes-related complications in the clusters (**a**, prevalence; **b**, incidence). See also Supplementary Table 2, Additional file 1 for numbers of cases in each cluster. *p < 0.05, **p < 0.01, ***p < 0.001 for comparisons between each cluster to cluster 2 (reference)

Figure 3a and Supplementary Table 2, Additional file 1 show the prevalence of CKD, DSPN and CVD among the clusters. There was a significant difference in the prevalence of all three complications between the clusters (*p* ≤ 0.0450). In adjusted pairwise comparisons with cluster 2 as reference (Supplementary Table 4, Additional file 1), CKD prevalence was elevated in clusters 3, 4, 5 and 6. These differences remained significant after adjustment for age, sex and BMI (all *p*_BH_ < 0.05). For DSPN, clusters did not differ significantly from cluster 2 but prevalences were 2–4 fold higher in all clusters compared with cluster 1. CVD prevalence was highest in clusters 1, 3 and 6. These differences were maintained after adjustment for age and sex (*p*_BH_ < 0.05), and the difference between cluster 3 and 2 remained significant after additional adjustment for BMI.

### Incidence of (pre)diabetes-related complications and all-cause mortality among clusters

Figure 3b and Supplementary Table 2, Additional file 1 illustrate the incidence of CKD and DSPN among the clusters. Significant differences were observed for CKD (*p* = 0.0487) but not for DSPN (*p* = 0.1322). Using cluster 2 as reference (Supplementary Table 5, Additional file 1), CKD incidence was higher in cluster 1, and DSPN incidence was higher in cluster 5.

Supplementary Fig. 2, Additional file 1 gives Kaplan–Meier curves for incident CVD and all-cause mortality which indicate only small between-cluster differences during the follow-up. CVD incidence was higher in cluster 3 compared to cluster 2 (age- and sex-adjusted p < 0.05; Supplementary Table 6, Additional file 1) but this difference was not significant after adjustment for multiple testing or BMI. No significant differences were seen for all-cause mortality.

### Comparison of the inflammatory load between the clusters

Supplementary Fig. 3, Additional file 1 displays the variable loading plot of the PCA including the 73 biomarkers of inflammation. Since principal component 1 was the only highly stable component after bootstrapping (Supplementary Fig. 4, Additional file 1), we calculated the inflammatory load for each participant based on principal component 1 only. The individual loadings of the biomarkers to principal component 1 are given in Supplementary Table 7, Additional file 1. Correlations between the inflammatory load and the clustering variables are described in Supplementary Table 8, Additional file 1.

**Fig. 4:**
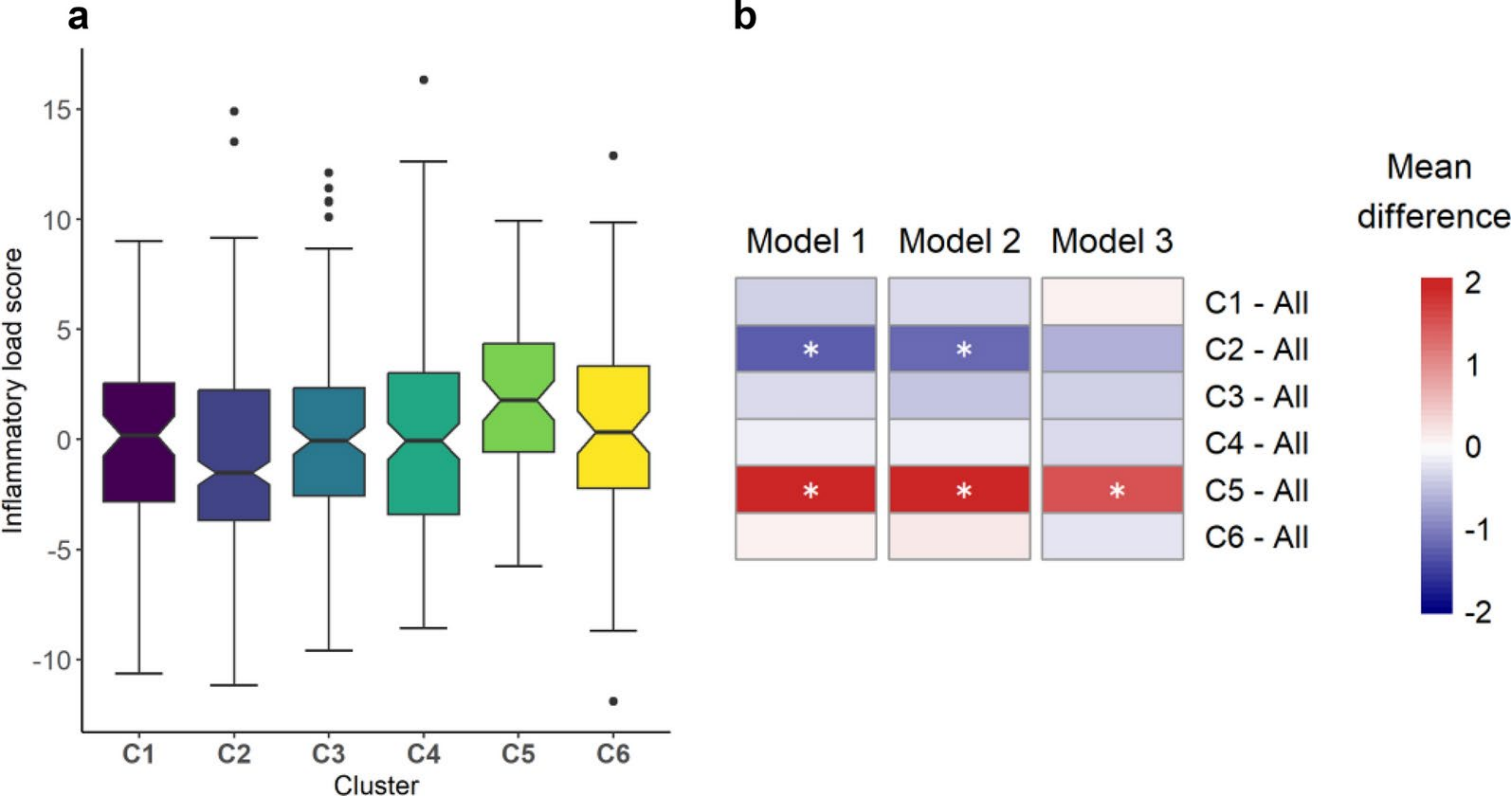
Comparison of the inflammatory load between the clusters. **a** Notched boxplots of the inflammatory load (PC score) for each cluster. The lower whisker indicates the smallest observation ≥ lower hinge-1.5*IQR. The upper whisker indicates the largest observation ≤ upper hinge + 1.5*IQR. **b** Pairwise comparison of the inflammatory load (PC score) between each cluster against all other clusters combined. Model 1, unadjusted; model 2, adjusted for age and sex; model 3, model 2 + BMI. The color of the heatmap indicates the difference in mean levels of the groups and the asterisks (*) indicate the significance (BH corrected *p* < 0.05). All *p* values were adjusted with Benjamini–Hochberg correction for all 6 comparisons. Clusters are named “C1”, “C2”, “C3”, “C4”, “C5”, and “C6”. PC score: principal component score

Participants in cluster 5 had the highest inflammatory load, while participants in cluster 2 had the lowest (Fig. 4a). Pairwise comparisons further demonstrated that cluster 5 had a significantly higher inflammatory load compared to all other clusters combined after adjustment for age, sex, and BMI (Fig. 4b). When compared to each individual cluster, cluster 5 had a higher inflammatory load than clusters 1, 2, 3, and 4 in age and sex-adjusted analysis (Supplementary Fig. 5, Additional file 1). Cluster 2 had a lower inflammatory load than the other clusters combined when adjusted for age and sex, but the difference became non-significant after additionally adjusting for BMI (Fig. 4b). All other clusters had intermediate values of the inflammatory load (Fig. 4b, Supplementary Fig. 5, Additional file 1).

In order to investigate to what extent between-cluster differences in prevalent and incident complications might be explained by subclinical inflammation, we additionally adjusted for the inflammatory load. Differences in CKD prevalence were attenuated compared to the age- and sex-adjusted model but were similar to the model adjusted for age, sex and BMI. Adjustment for the inflammatory load had almost no effect on differences in prevalent DSPN and CVD compared to the age- and sex-adjusted model (Supplementary Table 4, Additional file 1**)**. Additional adjustment for the inflammatory load attenuated the differences between clusters 5 and 2 for incident DSPN, but had virtually no effect on effect estimates describing between-cluster differences in incident CKD, T2D, CVD or all-causemortality (Supplementary Tables 3, 5, 6, Additional file 1).

### Comparison of biomarkers of subclinical inflammation between the clusters

**Fig. 5:**
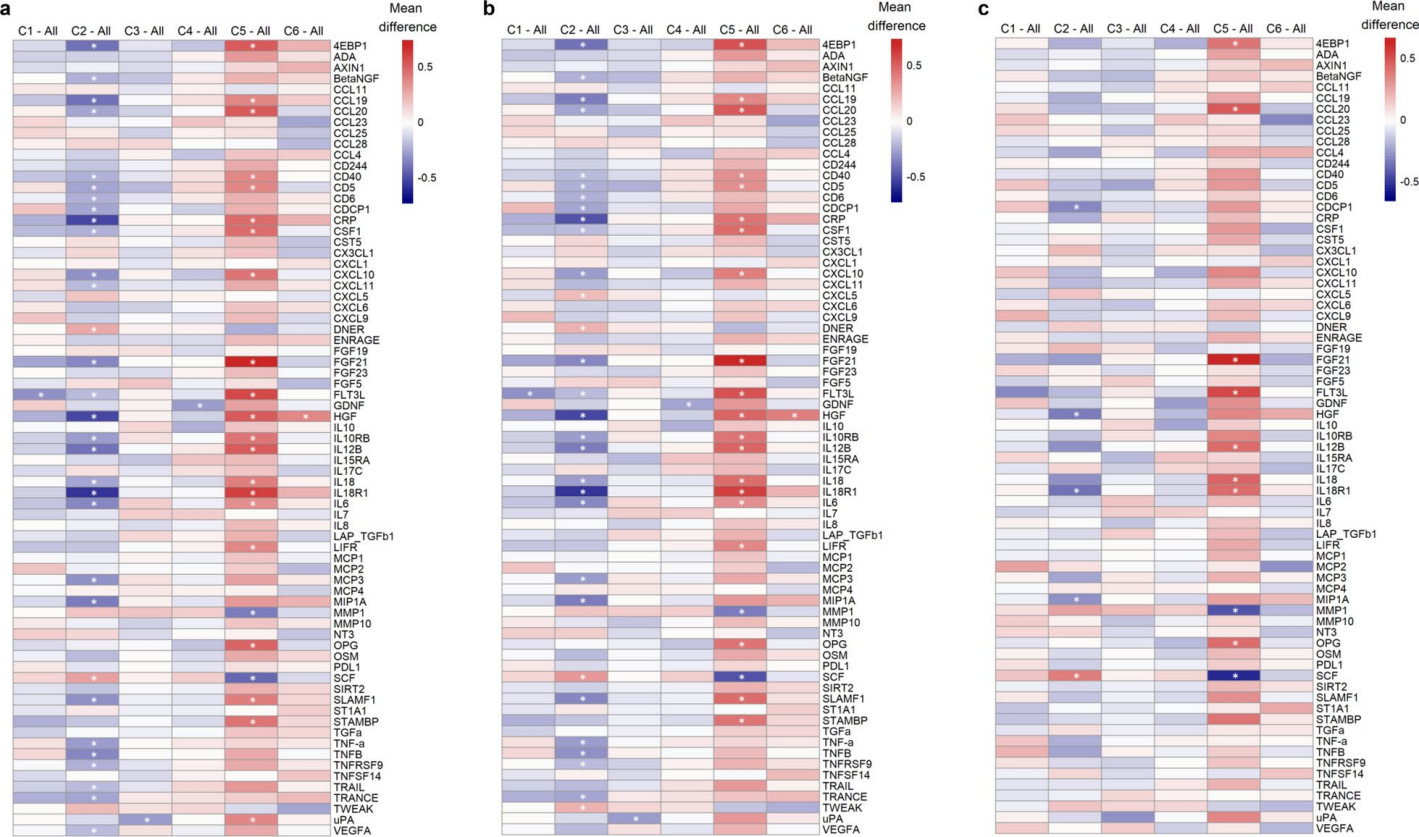
Pairwise comparisons of biomarkers of inflammation between each cluster against all other clusters combined. **a** unadjusted; **b** adjusted for age and sex; **c**, adjusted for age, sex, and BMI. The colour of the heatmap indicates the difference in mean levels between the groups and the asterisks (*) indicate statistical significance (*p*_BH_ < 0.05). All *p* values were adjusted with Benjamini–Hochberg correction for all 438 tests (73 markers and 6 comparisons). Clusters are named “C1”, “C2”, “C3”, “C4”, “C5”, and “C6”

In exploratory analyses, biomarkers of inflammation were analysed separately. As shown in Fig. 5a, most differences in biomarker levels were seen for cluster 2 (n = 31, of which 29 were lower than in the other clusters combined) and for cluster 5 (n = 23, of which 21 were higher than in the other clusters combined) with 18 biomarkers showing differences for both clusters 2 and 5 (in opposite directions) versus the other clusters combined. Clusters 1, 3, 4 and 6 differed only for one biomarker each from the other clusters. These findings were almost unchanged after adjustment for age and sex (Fig. 5b), whereas further adjustment for BMI (Fig. 5c) reduced the numbers of biomarker differences to 5 and 10 for clusters 2 and 5, respectively. Supplementary Fig. 6, Additional file 1 provides further details by summarising all pairwise comparisons (each cluster vs. each other cluster separately).

## Discussion

This study replicated the clusters described by Wagner et al. [9] in older adults from the population-based KORA F4/FF4 cohort and extended current knowledge by the analysis in an older population, by investigating DSPN as an outcome and by exploring numerous biomarkers of inflammation. Clusters differed (i) in their cardiometabolic risk profile, (ii) in T2D incidence, (iii) in the prevalence of CKD, DSPN and CVD, (iv) in the incidence of CKD and DSPN and (v) in their inflammatory load. The adjustment for inflammatory load had only a minor impact on the differences in (pre)diabetes-related complications between clusters.

### Replication of phenotype-based clusters before the diagnosis of diabetes

Our study represents a comprehensive replication of the phenotype-based clusters in people without T2D that were identified in the Tübingen Family study and Tübingen Lifestyle Intevention Program (TUEF/TULIP) cohort and the Whitehall II cohort [9]. Whereas TUEF/TULIP enrolled people at elevated risk of T2D, the occupational Whitehall II cohort comprised people with a lower risk of T2D, but importantly both cohorts mainly consisted of middle-aged people. Our study is the first replication that extends the analysis to older individuals (all > 60 years of age) with a high cardiometabolic risk.

Use of the same clustering algorithm yielded a similar distribution of glucose tolerance categories as in TUEF/TULIP: high prevalence of NGT in the low-risk clusters 1, 2 and 4 compared with a high prevalence of IGT and IFG/IGT in the high-risk clusters 3, 5 and 6. The comparability with the data from Whitehall II is limited because of the low proportion of people with IGT and IFG/IGT in this cohort. The radar charts in KORA F4 display similar differences in the overall cardiometabolic risk profile between the clusters as in TUEF/TULIP and in Whitehall II with the most favourable profile in clusters 1 and 2 and the most unfavourable profile in clusters 5 and 6. Therefore, these findings indicate that the phenotype-based clustering from Wagner et al. can identify subgroups in people without T2D with different cardiometabolic risk profile in populations with a wide range of mean age and overall T2D risk.

The six clusters showed pronounced differences in the incidence of T2D. The highest T2D incidence was observed for clusters 5 and 3, which is in line with the data from TUEF/TULIP and Whitehall II [9]. T2D risk was higher in clusters 6 and 4 compared with cluster 2 (lowest risk), which indicates a further risk separation between the cluster 4 and the low risk clusters 1 and 2 in KORA. These between-cluster differences were robust to adjustment for age and sex but slightly attenuated when we additionally adjusted for BMI.

Our study shows that the six clusters also differed in the prevalence of CKD, DSPN and CVD. DSPN has not been investigated in this context before. We found the nominally lowest prevalence of DSPN in cluster 1, and 2–4 fold higher prevalences in the other clusters albeit differences did not reach statistical significance. Nonetheless, these findings point towards a clinically relevant heterogeneity in DSPN risk among people without T2D. They also indicate differences between DSPN on the one hand and CKD and CVD on the other hand, which are characterised by the lowest prevalence in cluster 2. With respect to CKD, our results show robust differences between the clusters with the lowest prevalence in cluster 2 and ORs between 2 and 4 for the other clusters compared to cluster 2. This suggests that the clustering might be more sensitive to identify differences in renal function in older people, whereas the six clusters did not differ in urinary albumin/creatinine ratio in the younger TUEF/TULIP study participants. Regarding CVD, clusters 1, 3 and 6 had the highest prevalence in KORA F4. The analyses in TUEF/TULIP used carotid intima-media thickness as a proxy for CVD, which was higher in clusters 3, 5 and 6 than in clusters 1, 2 and 4. Thus, at least clusters 3 and 6 appear to be characterised by elevated CV risk. Taken together, the cluster assignment can also be used in older people to address the heterogeneity in (pre)diabetes-related complications. The findings appeared most consistent for cluster 2 as the low-risk subgroup and clusters 3, 5 and 6 as the high-risk subgroups.

For incident CKD, DSPN and CVD, differences between the six clusters were less pronounced compared to the differences in their prevalence. The CKD incidence was highest in cluster 1, whereas CKD risk was low in this cluster in TUEF/TULIP and Whitehall II. DSPN risk, which had not been examined in previous studies, was highest in cluster 5. The low CVD risk in cluster 2 is in line with data from Whitehall II, but we found the highest CVD incidence in cluster 3 versus cluster 5 in Whitehall II. In people with coronary angiography from the LURIC study a higher CV risk was seen for clusters 3, 5 and 6 combined versus clusters 1, 2 and 4 [23]. While we observed a nominally higher CVD incidence in clusters 3 and 5 compared to clusters 2 and 4, our findings for clusters 1 and 6 do not fit this pattern. We did not see differences in mortality rate between clusters which might be explained by the higher mean age and the shorter follow-up time in KORA F4/FF4 compared to Whitehall II.

Taken together, the cluster assignment was very sensitive to reveal the heterogeneity in T2D risk in our older study population, whereas the risk stratification for incident CKD, DSPN, CVD and mortality was less effective. Of note, we are not aware of further studies that used the phenotype-based clustering from Wagner et al. A similar clustering approach, but based on different variables, was used in a large sample of people with prediabetes [24]. This study also identified six clusters but with smaller differences in the overall cardiometabolic risk profile than in KORA F4. Their prospective analysis showed that the clusters were useful for the stratification of T2D risk, whereas between-cluster differences regarding CKD or CVD risk were less pronounced, which is in line with our findings.

### Differences in subclinical inflammation between clusters

This study used a multimarker proteomics panel to characterise differences in subclinical inflammation between phenotype-based clusters. After dimensionality reduction we found that the inflammatory load was highest in cluster 5 previously designated as “high risk insulin resistant fatty liver”, whereas cluster 2 designated as “very low risk” had the lowest inflammatory load. In contrast, the inflammatory load was intermediate for clusters 1, 3, 4 and 6 with relatively few between-cluster differences.

The high inflammatory load in cluster 5 is in line with its clinical characteristics, i.e. obesity, high insulin resistance, high prevalence of hypertension and very high hepatic lipid content [9], and with the increased burden and risk of (pre)diabetes-related complications compared with the other clusters. Since parameters that were used for the clustering such as triglycerides and BMI were strongly correlated with the inflammatory load and had the highest levels in cluster 5, this might explain why the highest inflammatory load was observed in cluster 5. While conclusions on mechanistic or causal relationships are not possible based on our data, previous studies have linked multiple biomarkers or their receptors, that characterise the inflammatory signature of cluster 5, with the development of insulin resistance. These include components of the IL-6 family (IL-6, LIFR [25, 26]), cytokines downstream of the NLRP3 inflammasome (IL-18/IL-18R1; [27, 28]), chemokines (CCL19 [29]) and the CD40/CD40L signaling [30]. In this respect, cluster 5 may be antecedent to the SIRD subtype in people with diabetes which is also characterised by both high risk of complications [1, 2] and high subclinical inflammation [5, 6].

However, it is noteworthy that the high risk of T2D and complications in clusters 3 and 6 is not reflected by an elevated inflammatory load which suggests that different mechanisms may underlie the high-risk phenotype in the different clusters. It is possible that high oxidative stress may contribute to the higher risk in clusters 3 and 6, but mechanisms related to beta-cell dysfunction (in particular for cluster 3), to differences in adipose tissue distribution (e.g. for visceral, pancreatic and renal sinus fat) or mitochondrial function are also plausible and merit further investigation.

Overall, these findings considerably extend the data from the TUEF/TULIP cohort which only included CRP and found, in line with our results, that CRP levels were highest in cluster 5 and lowest in cluster 2 [9]. Of note, CRP only had a small contribution to the inflammatory load score of this study, suggesting that the differences between the clusters cannot be sufficiently explained by this general indicator of subclinical inflammation and that other biomarkers would be needed to identify the potential mechanisms and pathways.

### Strengths and limitations

Strengths of the study include the population-based design, the assessment of glucose-tolerance status with OGTTs, the investigation of (pre)diabetes-related complications both cross-sectionally and prospectively and the availability of proteomics data for biomarkers of inflammation, which enabled us to derive the inflammatory load but also to analyse multiple biomarkers separately.

The major limitation of the study is the fact that certain phenotypes and variables from the TUEF/TULIP cohort such as insulin secretion calculated from glucose and insulin levels at 0 and 30 min from an OGTT or liver and renal sinus fat were not available in KORA F4. However, our dataset contained all variables required for the cluster validation as performed in the Whitehall II cohort. The numbers of study participants with prevalent or incident (pre)diabetes-related complications in the separate clusters differed so that larger studies would be required to better detect cluster differences especially with respect to incident CVD and mortality. Longer follow-up times might be needed to assess the differences between clusters with respect to the incidence of complications and mortality more accurately. The study participants are from one region in Southern Germany and mainly of European ancestry so that results are not generalisable to the whole German population or to populations with different ancestry. Finally, the observational study design precludes any causal inferences.

## Conclusions

This study corroborated the previously identified six clusters derived from phenotype-based clustering in a population-based sample of older people without clinically or OGTT-diagnosed T2D. When data from cross-sectional and prospective analyses are assessed together, data are most consistent for cluster 2 as low-risk cluster and for clusters 3, 5 and 6 as high-risk clusters. However, the cluster assignment appears to capture phenotypic heterogeneity better for the risk of T2D than for its complications or mortality in an older population. This is the first study to implement a proteomics approach to derive a multimarker-based inflammatory load, which differed between the prediabetes clusters. The high inflammatory load in cluster 5 and the low inflammatory load in cluster 2 correspond to their overall cardiometabolic risk profile, whereas other pathways might underlie the high-risk phenotypes of clusters 3 and 6. The high inflammatory load and the high burden of complications in cluster 5 might identify a subgroup of people who could benefit from early prevention also addressing the high inflammatory state.

## Supplementary Information

Additional file 1.

## Data Availability

The datasets from this KORA study are not publicly available because the datasets are subject to national data protection laws, and restrictions were imposed by the ethics committee of the Bavarian Chamber of Physicians to ensure data privacy of the study participants. However, datasets are available on reasonable request through a project agreement from KORA (https://helmholtz-muenchen.managed-otrs.com/external/). Requests should be sent to kora.passt@helmholtz-munich.de and are subject to approval by the KORA board.

## Abbreviations

BMI: Body mass index
CI: Confidence interval
CKD: Chronic kidney disease
CVD: Cardiovascular disease
DSPN: Distal sensorimotor polyneuropathy
eGFR: Estimated glomerular filtration rate
HR: Hazard ratios
hsCRP: High-sensitivity C-reactive protein
IFG: Impaired fasting glucose
IGT: Impaired glucose tolerance
KORA: Cooperative Health Research in the Region of Augsburg
MARD: Mild age-related diabetes
MI: Myocardial infarction
MOD: Mild obesity-related diabetes
NGT: Normal glucose tolerance
NPX: Normalised protein expression
OGTT: Oral glucose tolerance test
OR: Odds ratio
PCA: Principal component analysis
SAID: Severe autoimmune diabetes
SIDD: Severe insulin-deficient diabetes
SIRD: Severe insulin-resistant diabetes
T1D: Type 1 diabetes
T2D: Type 2 diabetes
TGFb1: Transforming growth factor beta-1 proprotein
TNFα: Tumour necrosis factor alpha
TUEF/TULIP: Tübingen Family study/Tübingen Lifestyle Intervention Program
