# Supplementary material for: Phenotype-based clusters, inflammation and cardiometabolic complications in older people before the diagnosis of type 2 diabetes: KORA F4/FF4 cohort study
Source: Cardiovasc Diabetol. 2025 Feb 19;24:83. doi: 10.1186/s12933-025-02617-8 (PMC11841139; doi:10.1186/s12933-025-02617-8)
Supplement: Supplementary file 1 — Additional file 1. [file 12933_2025_2617_MOESM1_ESM.docx]

**Additional file 1**

**Supplementary Table 1.** Distribution of participants by glucose tolerance category and cluster

|  | **Cluster** | | | | | |  |
| --- | --- | --- | --- | --- | --- | --- | --- |
| **Glucose tolerance category (%)** | 1  Low risk | 2  Very low risk | 3  Beta-cell failure | 4  Low risk obese | 5  High risk IR fatty liver | 6  High risk visceral fat nephropathy | *p* |
| **NGT (%)** | 70.11 | 69.00 | 6.92 | 64.67 | 7.04 | 43.68 | **<0.0001** |
| **IGT (%)** | 8.05 | 7.03 | 33.33 | 6.00 | 18.31 | 5.75 |  |
| **IFG/IGT (%)** | 0 | 4.15 | 39.62 | 0 | 54.93 | 9.20 |  |
| **IFG (%)** | 21.84 | 19.80 | 20.13 | 29.33 | 19.72 | 41.38 |  |

IFG, impaired fasting glucose; IGT, impaired glucose tolerance; IR, insulin resistance; NGT, normal glucose tolerance.

**Supplementary Table 2.** (Pre)diabetes-related complications among the clusters

|  | **Complication** | **Total** | **N (%) with complication in total group** | **Cluster 1** | **Cluster 2** | **Cluster 3** | **Cluster 4** | **Cluster 5** | **Cluster 6** | ***p*** |
| --- | --- | --- | --- | --- | --- | --- | --- | --- | --- | --- |
| **Prevalence*** | **CKD (n, %)** | 864 | 105 | 11 (12.6) | 20 (6.4) | 22 (13.9) | 23 (15.3) | 14 (19.7) | 15 (17.4) | **0.0035** |
|  | **DSPN (n, %)** | 837 | 125 | 5 (6.0) | 39 (12.7) | 32 (20.5) | 24 (17.1) | 12 (17.7) | 13 (15.9) | **0.0450** |
|  | **CVD (n, %)** | 867 | 63 | 8 (9.2) | 11 (3.5) | 21 (13.2) | 10 (6.7) | 4 (5.6) | 9 (10.3) | **0.0037** |
| **Incidence**** | **T2D (n, %)** | 502 | 76 | 3 (5.4) | 6 (3.2) | 29 (33.3) | 9 (10.5) | 17 (53.1) | 12 (22.2) | **<0.0001** |
|  | **CKD (n, %)** | 465 | 99 | 17 (34.0) | 30 (16.3) | 23 (28.1) | 14 (18.2) | 7 (26.9) | 8 (17.4) | **0.0487** |
|  | **DSPN (n, %)** | 434 | 101 | 13 (24.1) | 31 (18.5) | 18 (25.7) | 19 (26.8) | 11 (42.3) | 9 (20.0) | 0.1322 |

*F4 (2006-2008): total n=867; n=3 missing values for CKD; n=30 missing values for DSPN

**FF4 (2013-2014): n= 121 participants did not participate in FF4; total n=746; n=281 missing values for CKD; n=312 missing values for DSPN.

Significant differences (*p*<0.05) are in bold.

**Supplementary Table 3.** Differences in the incidence of T2D between the six clusters (logistic regression).

|  | **Model 1** | | | **Model 2** | | | **Model 3** | | | **Model 4** | | |
| --- | --- | --- | --- | --- | --- | --- | --- | --- | --- | --- | --- | --- |
|  | OR (95% CI) | *p* | *p*_BH_ | OR (95% CI) | *p* | *p*_BH_ | OR (95% CI) | *p* | *p*_BH_ | OR (95% CI) | *p* | *p*_BH_ |
| **Incident type 2 diabetes (n=486)** | | | | | | | | | | | | |
| Cluster 1 | 1.706 (0.412, 7.061) | 0.4613 | 0.4613 | 1.735 (0.418, 7.191) | 0.4477 | 0.4477 | 1.558 (0.374, 6.489) | 0.5424 | 0.5424 | 1.758 (0.424, 7.293) | 0.4372 | 0.4372 |
| Cluster 2 | Ref. | Ref. | Ref. | Ref. | Ref. | Ref. | Ref. | Ref. | Ref. | Ref. | Ref. | Ref. |
| Cluster 3 | **13.463 (5.273, 34.373)** | **<0.0001** | **0.0002** | **14.228 (5.533, 36.585)** | **<0.0001** | **0.0002** | **11.165 (4.204, 29.655)** | **<0.0001** | **0.0003** | **14.340 (5.573, 36.900)** | **<0.0001** | **0.0002** |
| Cluster 4 | **3.389 (1.166, 9.854)** | **0.0250** | **0.0313** | **3.556 (1.218, 10.380)** | **0.0203** | **0.0254** | 2.292 (0.714, 7.360) | 0.1634 | 0.2043 | **3.589 (1.229, 10.483)** | **0.0195** | **0.0244** |
| Cluster 5 | **32.863 (11.272, 95.783)** | **<0.0001** | **0.0002** | **33.996 (11.592, 99.699)** | **<0.0001** | **0.0002** | **18.765 (5.499, 64.039)** | **<0.0001** | **0.0003** | **36.598 (12.086, 110.820)** | **<0.0001** | **0.0002** |
| Cluster 6 | **7.780 (2.719, 22.260)** | **0.0001** | **0.0002** | **7.862 (2.743, 22.535)** | **0.0001** | **0.0002** | **4.548 (1.362, 15.185)** | **0.0138** | **0.0230** | **8.060 (2.801, 23.195)** | **0.0001** | **0.0002** |

Model 1: Unadjusted.

Model 2: Adjusted for age and sex.

Model 3: Adjusted for age, sex, and BMI.

Model 4: Adjusted for age, sex, and inflammatory load.

**Supplementary Table 4.** Differences in the prevalence of (pre)diabetes-related complications between the six clusters (logistic regression).

|  | **Model 1** | | | **Model 2** | | | **Model 3** | | | **Model 4** | | |
| --- | --- | --- | --- | --- | --- | --- | --- | --- | --- | --- | --- | --- |
|  | OR (95% CI) | *p* | *p*_BH_ | OR (95% CI) | *p* | *p*_BH_ | OR (95% CI) | *p* | *p*_BH_ | OR (95% CI) | *p* | *p*_BH_ |
| **Prevalent CKD (n=843)** | | | | | | | | | | | | |
| Cluster 1 | 2.007 (0.896, 4.497) | 0.0906 | 0.0906 | 2.142 (0.908, 5.053) | 0.0818 | 0.0818 | 2.111 (0.894, 4.984) | 0.0883 | 0.0883 | 2.062 (0.842, 5.048) | 0.1131 | 0.1131 |
| Cluster 2 | Ref. | Ref. | Ref. | Ref. | Ref. | Ref. | Ref. | Ref. | Ref. | Ref. | Ref. | Ref. |
| Cluster 3 | **2.547 (1.333, 4.869)** | **0.0047** | **0.0059** | **2.349 (1.180, 4.672)** | **0.0150** | **0.0188** | **2.173 (1.046, 4.515)** | **0.0375** | **0.0469** | **2.199 (1.067, 4.532)** | **0.0327** | **0.0409** |
| Cluster 4 | **2.792 (1.468, 5.311)** | **0.0018** | **0.0030** | **3.152 (1.579, 6.292)** | **0.0011** | **0.0018** | **2.808 (1.283, 6.148)** | **0.0098** | **0.0178** | **2.666 (1.283, 5.540)** | **0.0086** | **0.0160** |
| Cluster 5 | **3.763 (1.782, 7.945)** | **0.0005** | **0.0025** | **4.144 (1.851, 9.280)** | **0.0005** | **0.0013** | **3.512 (1.338, 9.218)** | **0.0107** | **0.0178** | **3.043 (1.311, 7.066)** | **0.0096** | **0.0160** |
| Cluster 6 | **3.272 (1.583, 6.764)** | **0.0014** | **0.0030** | **4.195 (1.918, 9.174)** | **0.0003** | **0.0013** | **3.555 (1.379, 9.164)** | **0.0087** | **0.0178** | **3.837 (1.722, 8.551)** | **0.0010** | **0.0050** |
| **Prevalent DSPN (n=814)** | | | | | | | | | | | | |
| Cluster 1 | 0.433 (0.165, 1.137) | 0.0892 | 0.2230 | 0.430 (0.162, 1.145) | 0.0912 | 0.3585 | 0.406 (0.153, 1.081) | 0.0713 | 0.3565 | 0.417 (0.157, 1.110) | 0.0801 | 0.4005 |
| Cluster 2 | Ref. | Ref. | Ref. | Ref. | Ref. | Ref. | Ref. | Ref. | Ref. | Ref. | Ref. | Ref. |
| Cluster 3 | 1.661 (0.951, 2.729) | 0.0761 | 0.2230 | 1.487 (0.865, 2.556) | 0.1511 | 0.3585 | 1.087 (0.605, 1.953) | 0.7815 | 0.7815 | 1.426 (0.827, 2.459) | 0.2022 | 0.5055 |
| Cluster 4 | 1.345 (0.768. 2.356) | 0.2998 | 0.3758 | 1.428 (0.802, 2.544) | 0.2262 | 0.3585 | 0.890 (0.457, 1.734) | 0.7320 | 0.7815 | 1.341 (0.748, 2.404) | 0.3239 | 0.5398 |
| Cluster 5 | 1.455 (0.716, 2.957) | 0.3006 | 0.3758 | 1.486 (0.717, 3.082) | 0.2868 | 0.3585 | 0.762 (0.319, 1.817) | 0.5393 | 0.7815 | 1.290 (0.614, 2.707) | 0.5014 | 0.5442 |
| Cluster 6 | 1.294 (0.654, 2.560) | 0.4600 | 0.4600 | 1.322 (0.657, 2.660) | 0.4338 | 0.4338 | 0.661 (0.278, 1.574) | 0.3496 | 0.7815 | 1.243 (0.616, 2.508) | 0.5442 | 0.5442 |
| **Prevalent CVD (n=843)** | | | | | | | | | | | | |
| Cluster 1 | **2.776 (1.080, 7.141)** | **0.0341** | 0.0568 | **2.969 (1.144, 7.706)** | **0.0254** | **0.0423** | **2.925 (1.125, 7.603)** | **0.0277** | 0.0693 | **2.909 (1.119, 7.558)** | **0.0284** | **0.0473** |
| Cluster 2 | Ref. | Ref. | Ref. | Ref. | Ref. | Ref. | Ref. | Ref. | Ref. | Ref. | Ref. | Ref. |
| Cluster 3 | **4.049 (1.887, 8.692)** | **0.0003** | **0.0015** | **4.021 (1.859, 8.701)** | **0.0004** | **0.0020** | **3.725 (1.636, 8.480)** | **0.0017** | **0.0075** | **3.962 (1.829, 8.581)** | **0.0005** | **0.0025** |
| Cluster 4 | 1.951 (0.809, 4.703) | 0.1367 | 0.1709 | 2.136 (0.878, 5.198) | 0.0942 | 0.1178 | 1.904 (0.710, 5.110) | 0.2008 | 0.2510 | 2.075 (0.850, 5.062) | 0.1087 | 0.1359 |
| Cluster 5 | 1.620 (0.500, 5.245) | 0.4213 | 0.4213 | 1.800 (0.550, 5.895) | 0.3315 | 0.3315 | 1.521 (0.397, 5.832) | 0.5404 | 0.5404 | 1.648 (0.497, 5.463) | 0.4141 | 0.4141 |
| Cluster 6 | **3.207 (1.282, 8.020)** | **0.0127** | **0.0318** | **3.271 (1.296, 8.252)** | **0.0121** | **0.0303** | 2.771 (0.905, 8.486) | 0.0744 | 0.1240 | **3.158 (1.248, 7.991)** | **0.0152** | **0.0380** |

Model 1: Unadjusted

Model 2: Adjusted for age and sex

Model 3: Adjusted for age, sex, and BMI

Model 4: Adjusted for age, sex, and inflammatory load.

**Supplementary Table 5.** Differences in the incidence of CKD and DSPN between the six clusters (logistic regression).

|  | **Model 1** | | | **Model 2** | | | **Model 3** | | | **Model 4** | | |
| --- | --- | --- | --- | --- | --- | --- | --- | --- | --- | --- | --- | --- |
|  | OR (95% CI) | *p* | *p*_BH_ | OR (95% CI) | *p* | *p*_BH_ | OR (95% CI) | *p* | *p*_BH_ | OR (95% CI) | *p* | *p*_BH_ |
| **Incident CKD (n=452)** | | | | | | | | | | | | |
| Cluster 1 | **2.730 (1.342, 5.552)** | **0.0056** | **0.0140** | **2.800 (1.305, 6.006)** | **0.0082** | **0.0410** | **2.689 (1.246, 5.802)** | **0.0117** | 0.0585 | **2.801 (1.300, 6.034)** | **0.0085** | **0.0425** |
| Cluster 2 | Ref. | Ref. | Ref. | Ref. | Ref. | Ref. | Ref. | Ref. | Ref. | Ref. | Ref. | Ref. |
| Cluster 3 | **2.005 (1.090, 3.877)** | **0.0261** | 0.0653 | 1.861 (0.952, 3.638) | 0.0694 | 0.1735 | 1.670 (0.813, 3.430) | 0.1628 | 0.4070 | 1.841 (0.941, 3.604) | 0.0749 | 0.1873 |
| Cluster 4 | 1.142 (0.566, 2.305) | 0.7115 | 0.8109 | 1.276 (0.611, 2.667) | 0.5167 | 0.5614 | 1.052 (0.439, 2.521) | 0.9100 | 0.9530 | 1.243 (0.592, 2.609) | 0.5659 | 0.6908 |
| Cluster 5 | 1.893 (0.729, 4.912) | 0.1896 | 0.3160 | 2.142 (0.788, 5.828) | 0.1356 | 0.2260 | 1.619 (0.482, 5.438) | 0.4353 | 0.7255 | 1.782 (0.639, 4.970) | 0.2694 | 0.4490 |
| Cluster 6 | 1.111 (0.469, 2.629) | 0.8109 | 0.8109 | 1.304 (0.532, 3.197) | 0.5614 | 0.5614 | 1.033 (0.355, 3.007) | 0.9530 | 0.9530 | 1.202 (0.486, 2.972) | 0.6908 | 0.6908 |
| **Incident DSPN (n=419)** | | | | | | | | | | | | |
| Cluster 1 | 1.583 (0.749, 3.346) | 0.2288 | 0.2971 | 1.514 (0.707, 3.244) | 0.2859 | 0.3705 | 1.328 (0.614, 2.874) | 0.4709 | 0.7848 | 1.490 (0.689, 3.223) | 0.3114 | 0.4155 |
| Cluster 2 | Ref. | Ref. | Ref. | Ref. | Ref. | Ref. | Ref. | Ref. | Ref. | Ref. | Ref. | Ref. |
| Cluster 3 | 1.520 (0.759, 3.046) | 0.2377 | 0.2971 | 1.458 (0.718, 2.961) | 0.2964 | 0.3705 | 1.073 (0.500, 2.303) | 0.8556 | 0.9130 | 1.425 (0.696, 2.918) | 0.3324 | 0.4155 |
| Cluster 4 | 1.736 (0.893, 3.375) | 0.1041 | 0.2603 | 1.860 (0.945, 3.663) | 0.0726 | 0.1815 | 1.049 (0.448, 2.455) | 0.9130 | 0.9130 | 1.815 (0.913, 3.606) | 0.0890 | 0.2225 |
| Cluster 5 | **3.483 (1.447, 8.384)** | **0.0054** | **0.0270** | **3.456 (1.411, 8.463)** | **0.0067** | **0.0335** | 1.714 (0.578, 5.083) | 0.3312 | 0.7848 | **2.660 (1.063, 6.657)** | **0.0366** | 0.1830 |
| Cluster 6 | 1.257 (0.543, 2.913) | 0.5932 | 0.5932 | 1.244 (0.531, 2.916) | 0.6158 | 0.6158 | 0.679 (0.246, 1.876) | 0.4555 | 0.7848 | 1.181 (0.499, 2.796) | 0.7045 | 0.7045 |

Model 1: Unadjusted

Model 2: Adjusted for age and sex

Model 3: Adjusted for age, sex, and BMI

Model 4: Adjusted for age, sex, and inflammatory load

**Supplementary Table 6.** Differences in incident CVD and all-cause mortality between the six clusters (Cox regression).

|  | **Model 1** | | | **Model 2** | | | **Model 3** | | | **Model 4** | | |
| --- | --- | --- | --- | --- | --- | --- | --- | --- | --- | --- | --- | --- |
|  | HR (95% CI) | *p* | *p*_BH_ | HR (95% CI) | *p* | *p*_BH_ | HR (95% CI) | *p* | *p*_BH_ | HR (95% CI) | *p* | *p*_BH_ |
| **Incident CVD (n=724)** | | | | | | | | | | | | |
| Cluster 1 | 1.542 (0.675, 3.522) | 0.3043 | 0.4368 | 1.584 (0.692, 3.627) | 0.2767 | 0.4612 | 1.512 (0.659, 3.468) | 0.3296 | 0.8240 | 1.593 (0.695, 3.650) | 0.2709 | 0.4515 |
| Cluster 2 | Ref. | Ref. | Ref. | Ref. | Ref. | Ref. | Ref. | Ref. | Ref. | Ref. | Ref. | Ref. |
| Cluster 3 | **2.076 (1.090, 3.957)** | **0.0263** | 0.1315 | **1.916 (1.003, 3.659)** | **0.0490** | 0.2450 | 1.542 (0.774, 3.074) | 0.2183 | 0.8240 | **1.921 (1.006, 3.670)** | **0.0480** | 0.2400 |
| Cluster 4 | 1.070 (0.484, 2.365) | 0.8679 | 0.8679 | 1.090 (0.491, 2.423) | 0.8317 | 0.8317 | 0.774 (0.320. 1.872) | 0.5704 | 0.9507 | 1.107 (0.497, 2.464) | 0.8035 | 0.8035 |
| Cluster 5 | 1.550 (0.619, 3.882) | 0.3494 | 0.4368 | 1.456 (0.579, 3.658) | 0.4247 | 0.5309 | 0.865 (0.290, 2.574) | 0.7939 | 0.9791 | 1.519 (0.595, 3.877) | 0.3818 | 0.4773 |
| Cluster 6 | 1.696 (0.742, 3.874) | 0.2103 | 0.4368 | 1.719 (0.752, 3.929) | 0.1989 | 0.4612 | 1.014 (0.358, 2.872) | 0.9791 | 0.9791 | 1.746 (0.762, 4.000) | 0.1873 | 0.4515 |
| **All-cause mortality (n=843)** | | | | | | | | | | | | |
| Cluster 1 | 0.892 (0.474, 1.679) | 0.7228 | 0.7235 | 0.871 (0.462, 1.643) | 0.6701 | 0.7751 | 0.877 (0.465, 1.655) | 0.6857 | 0.9158 | 0.840 (0.445, 1.585) | 0.5913 | 0.8829 |
| Cluster 2 | Ref. | Ref. | Ref. | Ref. | Ref. | Ref. | Ref. | Ref. | Ref. | Ref. | Ref. | Ref. |
| Cluster 3 | 1.090 (0.676, 1.757) | 0.7235 | 0.7235 | 0.933 (0.578, 1.505) | 0.7751 | 0.7751 | 0.973 (0.580, 1.630) | 0.9158 | 0.9158 | 0.912 (0.565, 1.472) | 0.7063 | 0.8829 |
| Cluster 4 | 1.117 (0.689, 1.812) | 0.6536 | 0.7235 | 1.112 (0.683, 1.812) | 0.6690 | 0.7751 | 1.189 (0.667, 2.118) | 0.5580 | 0.9158 | 1.037 (0.634, 1.695) | 0.8849 | 0.8849 |
| Cluster 5 | 0.826 (0.405, 1.648) | 0.5992 | 0.7235 | 0.786 (0.385, 1.607) | 0.5100 | 0.7751 | 0.863 (0.374, 1.987) | 0.7285 | 0.9158 | 0.678 (0.329, 1.397) | 0.2922 | 0.8829 |
| Cluster 6 | 0.811 (0.421, 1.561) | 0.5306 | 0.7235 | 0.838 (0.435, 1.614) | 0.5978 | 0.7751 | 0.915 (0.424, 1.973) | 0.8198 | 0.9158 | 0.800 (0.415, 1.542) | 0.5046 | 0.8829 |

Model 1: Unadjusted.

Model 2: Adjusted for age and sex.

Model 3: Adjusted for age, sex, and BMI.

Model 4: Adjusted for age, sex and inflammatory load.

**Supplementary Table 7.** Loadings and contributions of the biomarkers of subclinical inflammation to principal component 1.

| **Biomarker** | **Loadings** | **Contributions (%)** |
| --- | --- | --- |
| CSF1 | 0.190 | 3.600 |
| CD40 | 0.182 | 3.323 |
| TNFRSF9 | 0.172 | 2.950 |
| HGF | 0.165 | 2.719 |
| CD5 | 0.164 | 2.673 |
| IL15RA | 0.163 | 2.650 |
| IL10RB | 0.162 | 2.625 |
| LIFR | 0.154 | 2.364 |
| 4EBP1 | 0.151 | 2.273 |
| CD244 | 0.147 | 2.165 |
| CX3CL1 | 0.147 | 2.160 |
| PDL1 | 0.146 | 2.127 |
| MIP1A | 0.146 | 2.121 |
| SLAMF1 | 0.142 | 2.014 |
| STAMBP | 0.139 | 1.941 |
| MCP3 | 0.136 | 1.837 |
| BetaNGF | 0.135 | 1.825 |
| CXCL11 | 0.134 | 1.806 |
| OPG | 0.134 | 1.785 |
| CXCL9 | 0.131 | 1.719 |
| SIRT2 | 0.130 | 1.684 |
| CXCL6 | 0.126 | 1.593 |
| TGFa | 0.126 | 1.589 |
| FGF23 | 0.125 | 1.551 |
| uPA | 0.124 | 1.540 |
| IL12B | 0.122 | 1.495 |
| LAP_TGFb1 | 0.122 | 1.490 |
| CCL23 | 0.122 | 1.482 |
| CD6 | 0.120 | 1.436 |
| CDCP1 | 0.120 | 1.435 |
| IL10 | 0.119 | 1.418 |
| CXCL10 | 0.119 | 1.415 |
| ADA | 0.119 | 1.413 |
| VEGFA | 0.118 | 1.401 |
| CCL11 | 0.116 | 1.356 |
| CCL28 | 0.116 | 1.345 |
| GDNF | 0.115 | 1.324 |
| IL18 | 0.114 | 1.298 |
| IL18R1 | 0.112 | 1.254 |
| CXCL1 | 0.110 | 1.204 |
| TRAIL | 0.109 | 1.197 |
| MMP10 | 0.109 | 1.194 |
| CCL25 | 0.109 | 1.187 |
| ENRAGE | 0.108 | 1.176 |
| FGF5 | 0.108 | 1.167 |
| TNFSF14 | 0.106 | 1.134 |
| IL8 | 0.106 | 1.126 |
| MCP1 | 0.103 | 1.068 |
| MCP4 | 0.103 | 1.065 |
| OSM | 0.103 | 1.058 |
| TNFB | 0.101 | 1.013 |
| CCL4 | 0.097 | 0.932 |
| CCL19 | 0.096 | 0.922 |
| TWEAK | 0.095 | 0.911 |
| AXIN1 | 0.093 | 0.857 |
| CST5 | 0.092 | 0.850 |
| CCL20 | 0.091 | 0.828 |
| IL17C | 0.090 | 0.813 |
| IL6 | 0.087 | 0.755 |
| IL7 | 0.086 | 0.740 |
| FLT3L | 0.080 | 0.635 |
| NT3 | 0.078 | 0.613 |
| DNER | 0.071 | 0.502 |
| FGF21 | 0.063 | 0.397 |
| CRP | 0.062 | 0.384 |
| CXCL5 | 0.058 | 0.337 |
| MCP2 | 0.058 | 0.335 |
| ST1A1 | 0.057 | 0.330 |
| TRANCE | 0.057 | 0.325 |
| SCF | 0.053 | 0.279 |
| TNF-a | 0.044 | 0.193 |
| FGF19 | 0.038 | 0.143 |
| MMP1 | 0.036 | 0.130 |

**Supplementary Table 8.** Correlation between the inflammatory load and the clustering variables.

| **Clustering variables** | **Inflammatory load score** | | | | | |
| --- | --- | --- | --- | --- | --- | --- |
|  | Model 1 | | Model 2 | | Model 3 | |
|  | ρ | p_B-H_ | ρ | *p*_B-H_ | ρ | *p*_B-H_ |
| Age (years) | 0.20 | **<0.0001** | 0.20 | **<0.0001** | 0.19 | **<0.0001** |
| BMI | 0.15 | **0.0001** | 0.13 | **0.0002** | - | - |
| Waist circumference | 0.14 | **0.0002** | 0.14 | **0.0002** | 0.05 | 0.2342 |
| Hip circumference | 0.13 | **0.0002** | 0.12 | **0.0006** | 0.02 | 0.5744 |
| Glucose (fasting) | 0.06 | 0.0930 | 0.06 | 0.0786 | 0.02 | 0.5744 |
| Glucose (2 h) | 0.12 | **0.0010** | 0.09 | **0.0086** | 0.06 | 0.1166 |
| AUC Glucose | 0.12 | **0.0009** | 0.10 | **0.0055** | 0.06 | 0.1166 |
| Insulin (fasting) | 0.12 | **0.0010** | 0.12 | **0.0011** | 0.06 | 0.1166 |
| Insulin (2 h) | 0.15 | **0.0001** | 0.14 | **0.0002** | 0.09 | **0.0348** |
| Triglycerides | 0.09 | **0.0089** | 0.12 | **0.0009** | 0.08 | **0.0463** |
| HDL cholesterol | -0.16 | **<0.0001** | -0.18 | **<0.0001** | -0.15 | **0.0003** |
| Insulin secretion | 0.06 | 0.0930 | 0.06 | 0.0778 | 0.04 | 0.3102 |
| Insulin sensitivity | -0.14 | **0.0001** | -0.14 | **0.0002** | -0.08 | **0.0463** |

Model 1: unadjusted; model 2: adjusted for age and sex; model 3: model 2 + BMI.

Bold font indicates significance (p_BH_<0.05). All *p*-values were adjusted with Benjamini-Hochberg correction for all 12 correlations (for model 3, 11 correlations).

Spearman’s rank correlation was used to calculate the coefficient ρ.

Insulin secretion was calculated using Stumvoll’s first-phase insulin-secretion index. Insulin sensitivity was calculated using the Matsuda index.

AUC, area under the curve; BMI, body mass index; HDL, high-density lipoprotein.

**

**

**Supplementary Figure 1.** Flow chart describing the selection of the study population

**a**

**
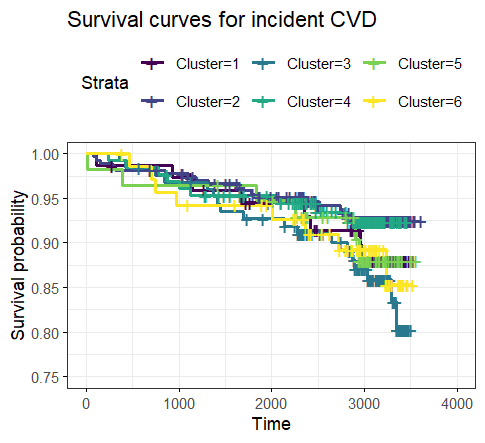
**

**b**


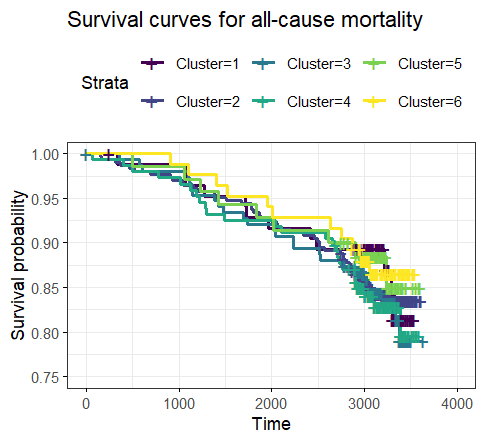


**Supplementary Figure 2.** Kaplan-Meier curves for the time to complication event for a) incident CVD and b) all-cause mortality for time in days. Please note the shortened range of the y-axis (from 0.75 to 1).


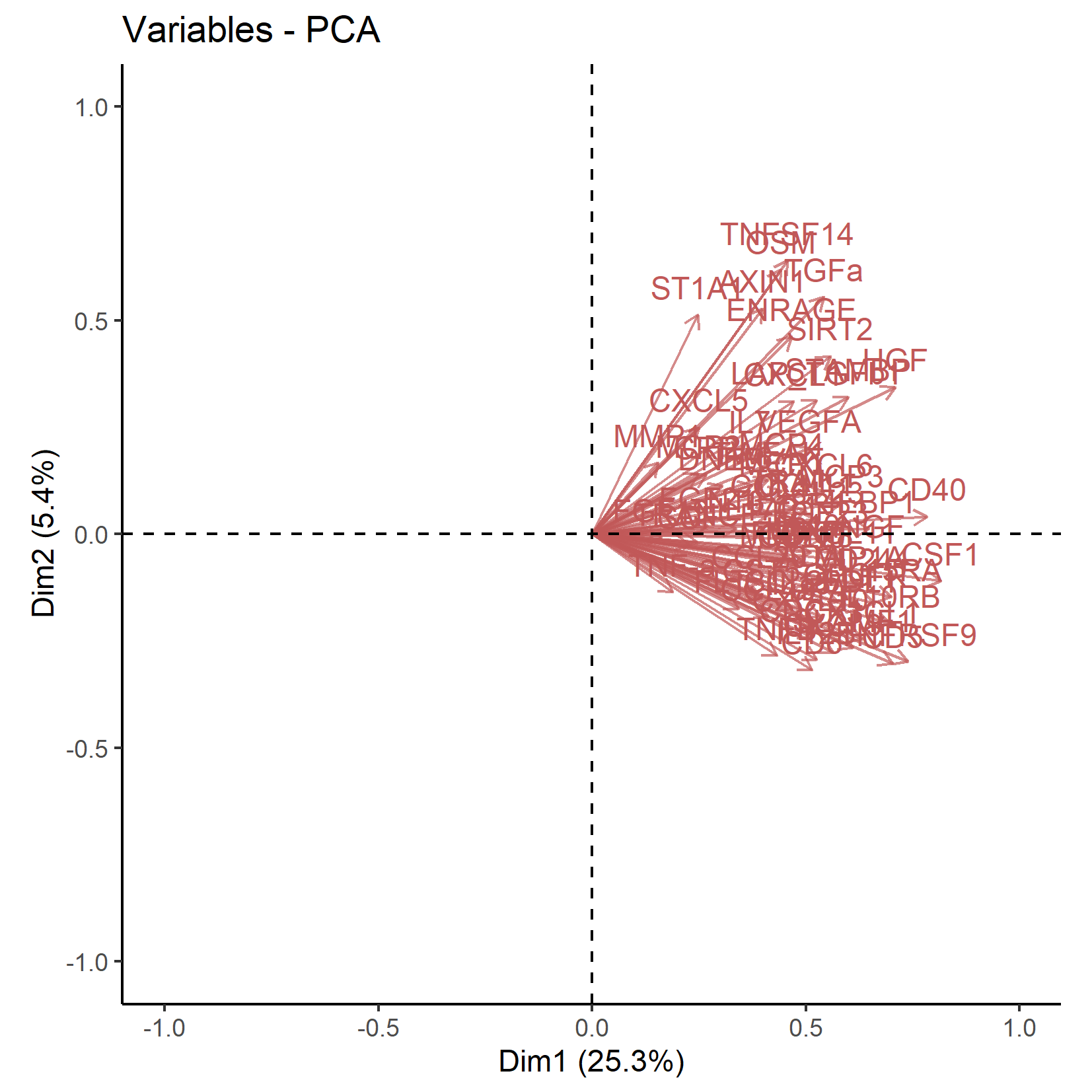


**Supplementary Figure 3.** Loading plot of the variables from the principal component analysis including 73 biomarkers of subclinical inflammation for principal components 1 and 2

The percentages of the dimensions on the axis titles indicate the percentages of explained variances.

Dim: Dimension (i.e., principal component).


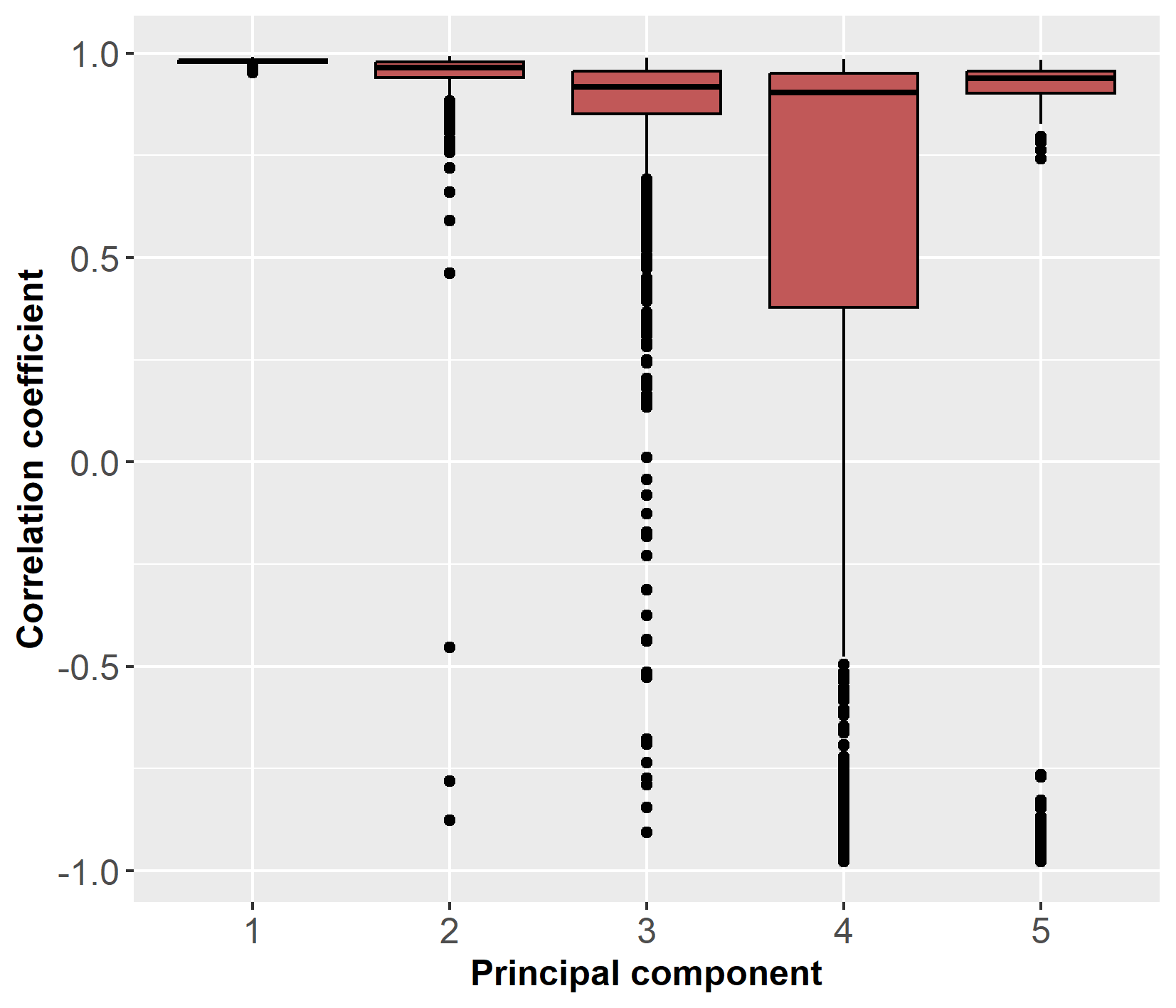


**Supplementary Figure 4.** Boxplots of Pearson correlation coefficients for the correlation between principal component variable scores of the original sample and 1000x bootstrap samples (comparison of the first five principal components)


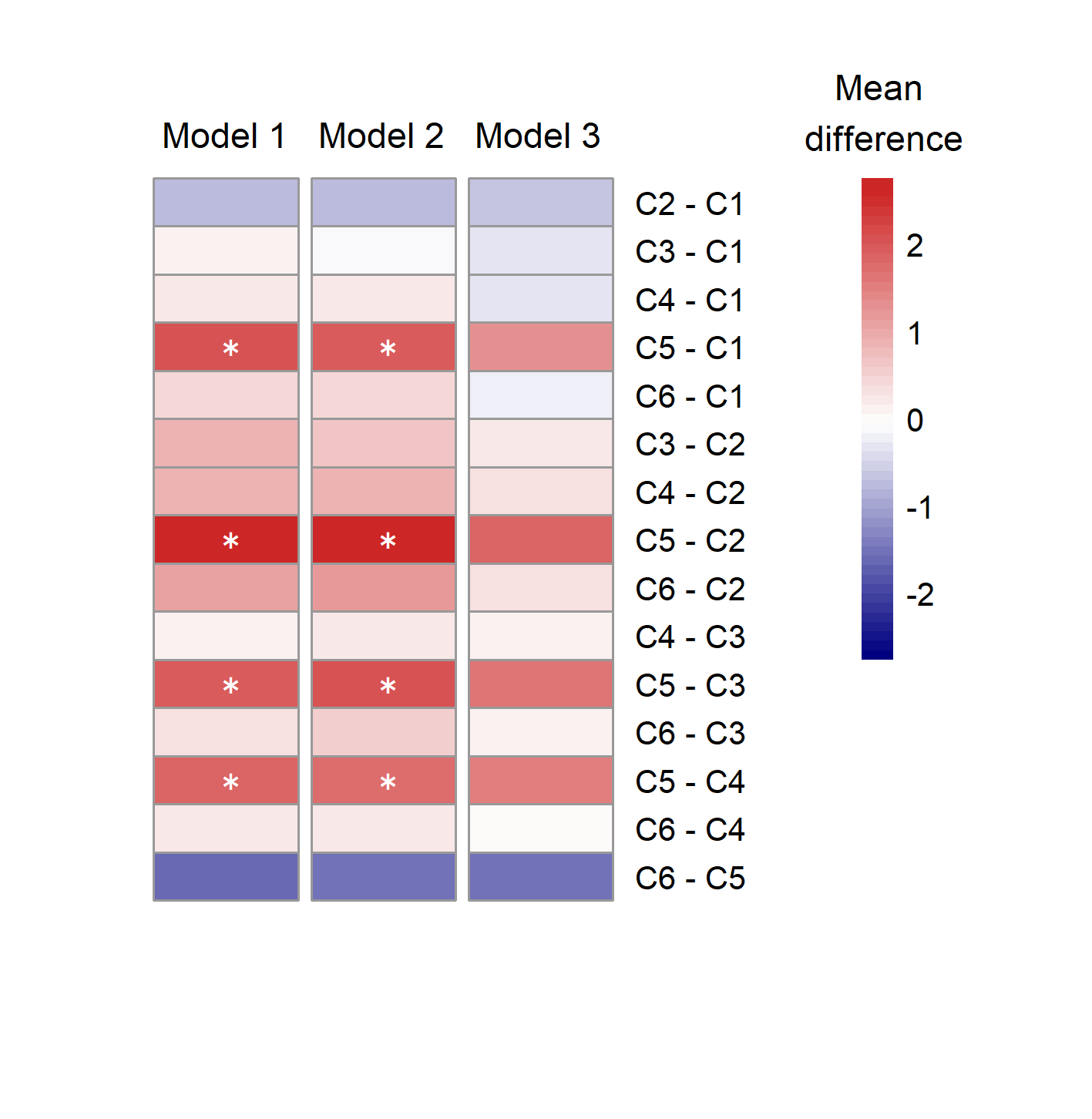


**Supplementary Figure 5.** Pairwise comparison of the inflammatory load (PC score) between all clusters

Model 1: unadjusted; model 2: adjusted for age and sex; model 3: model 2 + BMI. The colour of the heatmap indicates the difference in mean levels between the groups, and the asterisks (*) indicate the statistical significance (*p*_BH_<0.05). All *p*-values were adjusted with Benjamini-Hochberg correction for all 15 comparisons.

Clusters are named “C1”, “C2”, “C3”, “C4”, “C5”, and “C6”.


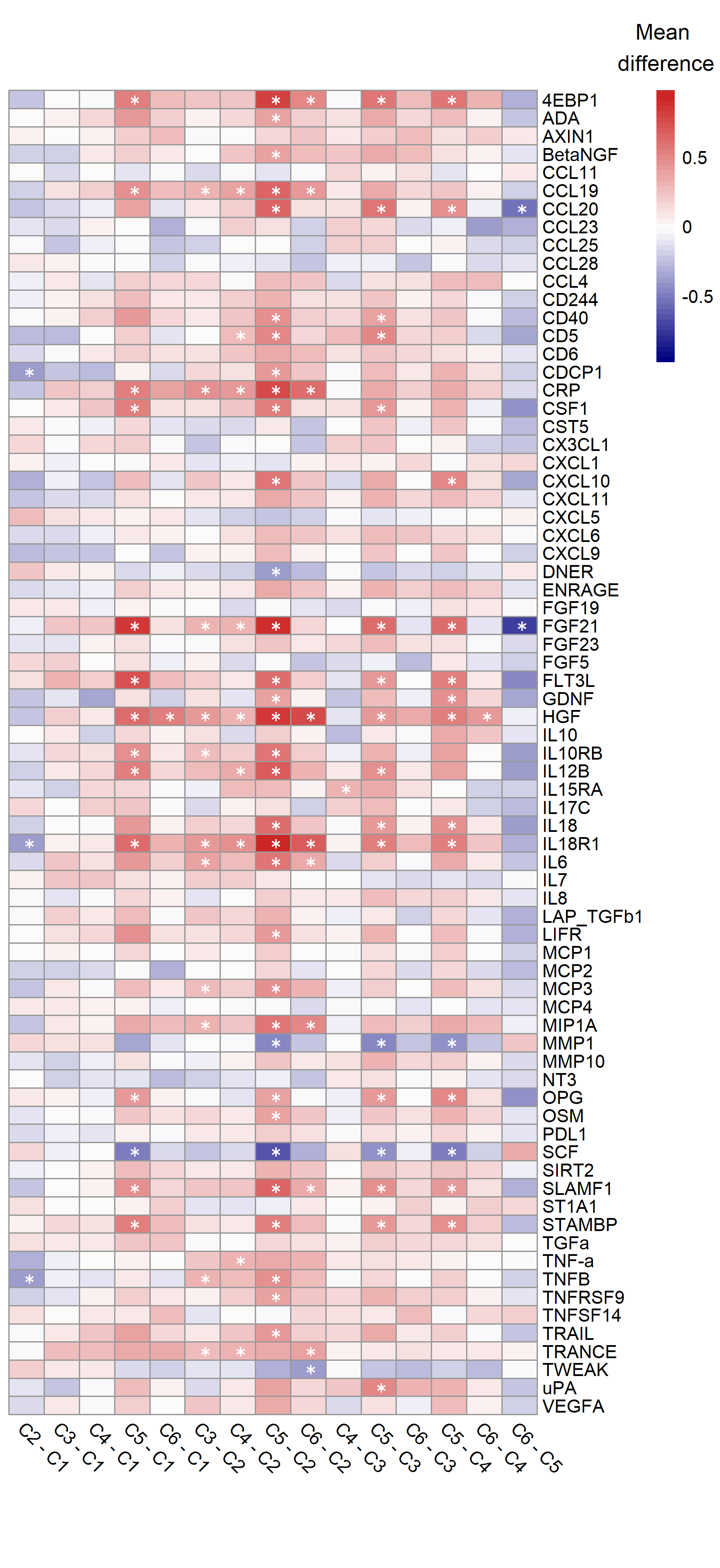

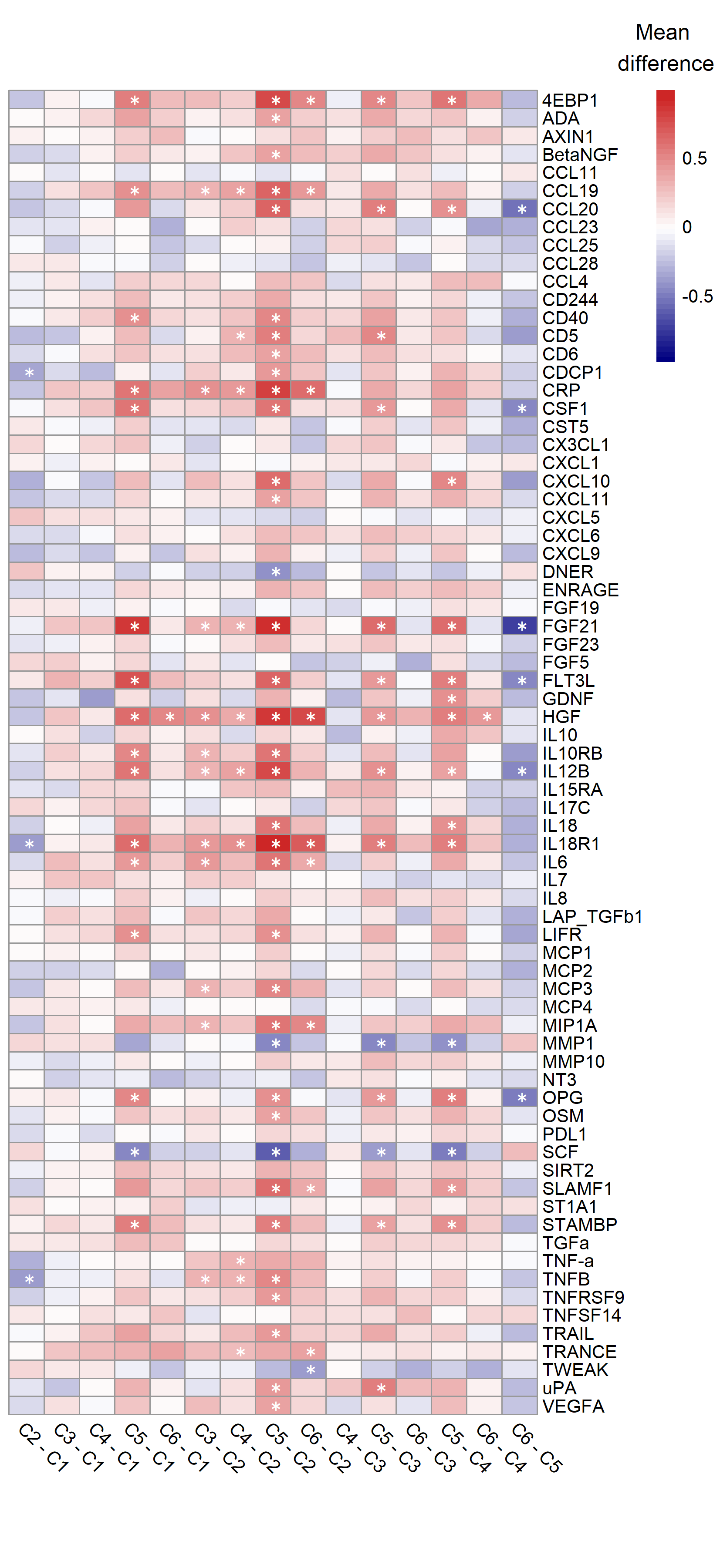

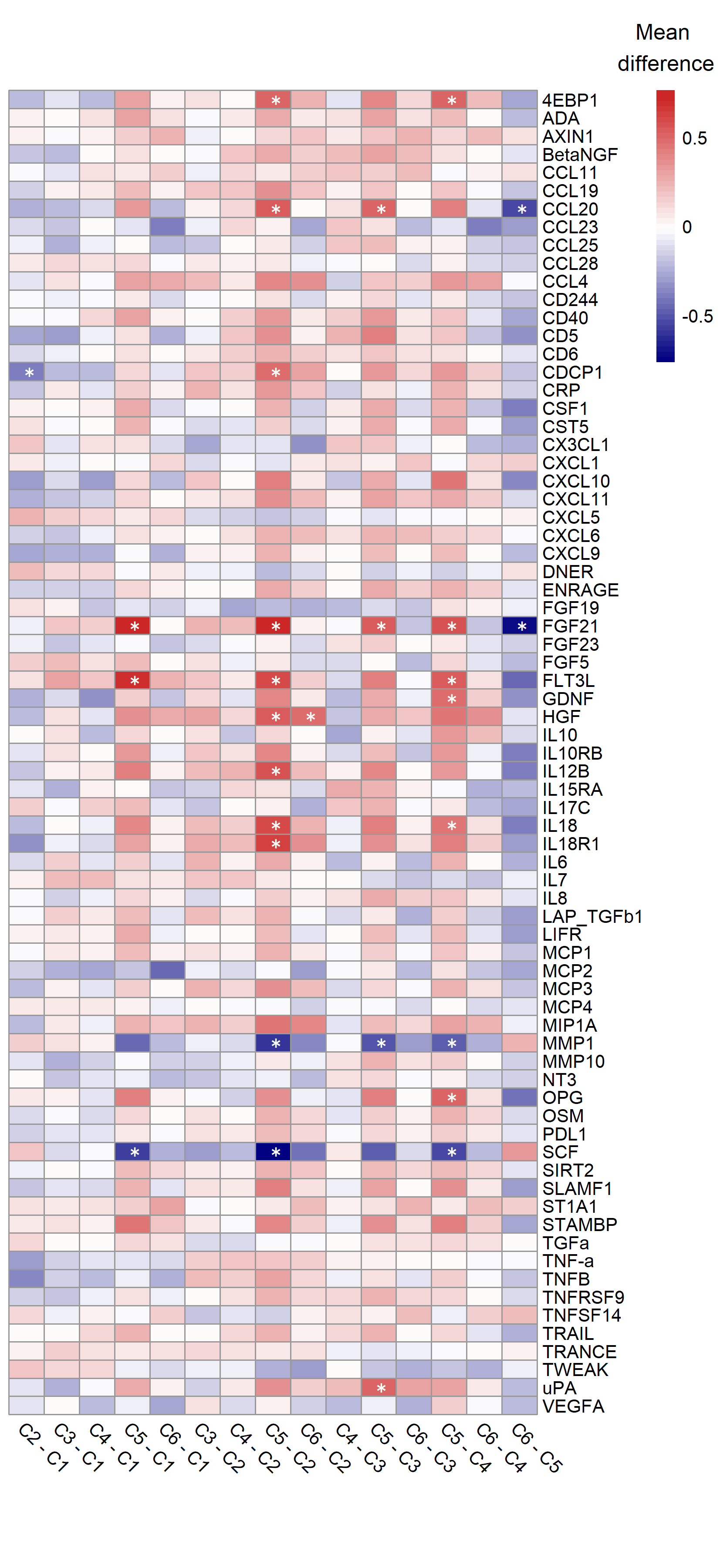


**c**

**b**

**a**

**Supplementary Figure 6.** Pairwise comparisons of biomarkers of inflammation between all clusters (a, unadjusted; b, adjusted for age and sex; c, adjusted for age, sex and BMI).

The colour of the heatmap indicates the difference in mean levels between the groups, and the asterisks (*) indicate the statistical significance (p_BH_<0.05).

All *p* values were adjusted with Benjamini-Hochberg correction for all 1095 tests (73 biomarkers and 15 comparisons).

Clusters are named “C1”, “C2”, “C3”, “C4”, “C5”, and “C6”.
